# Adherence to the 2018 World Cancer Research Fund/American Institute for Cancer Research Cancer Prevention Recommendations and risk of lifestyle-related cancers in the prostate, lung, colorectal, and ovarian cancer screening trial

**DOI:** 10.1038/s44276-025-00195-6

**Published:** 2025-11-19

**Authors:** Fiona C. Malcomson, Marissa M. Shams-White, Jill Reedy, Wen-Yi Huang, Steven C. Moore, Erikka Loftfield

**Affiliations:** 1 Metabolic Epidemiology Branch, Division of Cancer Epidemiology and Genetics, National Cancer Institute (NCI), Rockville, MD USA; 2 Human Nutrition & Exercise Research Centre, Centre for Healthier Lives, Population Health Sciences Institute, Newcastle University, Newcastle upon Tyne, UK; 3 Centre for Cancer, Population Health Sciences Institute, Newcastle University, Newcastle upon Tyne, UK; 4 Population Science, American Cancer Society, Atlanta, GA USA; 5 Risk Factor Assessment Branch, Division of Cancer Control and Population Sciences, NCI, Rockville, MD USA

## Abstract

**Background:**

The 2018 World Cancer Research Fund (WCRF)/American Institute for Cancer Research (AICR) Cancer Prevention Recommendations aim to lower cancer incidence. Our study explored associations between adherence to these Recommendations and the risk of first cancer incidence in a US cohort.

**Methods:**

Prostate, Lung, Colorectal and Ovarian (PLCO) Cancer Screening Trial Study participants who were cancer-free at baseline and who had height, weight, physical activity, diet, and alcohol data to estimate the standardized 2018 WCRF/AICR Score (*n* = 69,061) were included. Associations between Score and cancer endpoints, including any cancers, any of the 17 WCRF/AICR-reviewed cancers, and individual cancer sites were investigated using multivariable Cox proportional hazard models, adjusting for covariates.

**Results:**

Mean Score was 3.55 points (range 0–7). During a median follow-up of 10 years, 11,109 participants developed cancer. The 2018 WCRF/AICR Score was significantly inversely associated with risk of any cancers (HR per 1-point increment:0.97; 95% CI: 0.95–0.99), any of the 17 WCRF/AICR-reviewed cancers (HR: 0.97; 95% CI: 0.95–0.99), pancreatic (HR:0.86; 95% CI: 0.78–0.96), and breast (HR:0.91; 95% CI: 0.87–0.96) cancers.

**Discussion:**

Our findings support adherence to the WCRF/AICR Cancer Prevention Recommendations for reducing risk of cancer incidence, particularly breast and pancreatic cancers, in the US.

**Clinical registration numbers:**

The PLCO Cancer Screening Trial is registered with ClinicalTrials.gov: NCT00002540 (Prostate), NCT01696968 (Lung), NCT01696981 (Colorectal), NCT01696994 (Ovarian), and NCT00339495 (EEMS).

## Background

The World Cancer Research Fund (WCRF)/American Institute for Cancer Research (AICR) released ten evidence-based lifestyle recommendations in 2018 to reduce the risk of cancer worldwide [1]. Subsequently, the 2018 WCRF/AICR Score was a standardized scoring system created to examine alignment with recommendations focused on body weight, physical activity, dietary intake, alcohol intake, and breastfeeding. Researchers can utilize the score to investigate how adherence to the WCRF/AICR recommendations is associated with cancer-related outcomes [2, 3]. The remaining two WCRF/AICR Recommendations, not included in the score due to operational redundancy, are ‘do not use supplements for cancer prevention’ and ‘after a cancer diagnosis, follow our Recommendations, if you’re able to’ [1]. Dietary supplements are not recommended for cancer prevention and it is recommended that nutritional needs are met through diet alone [1].

Since publication of the 2018 WCRF/AICR Cancer Prevention Recommendations and creation of the 2018 WCRF/AICR Score, several studies have explored associations between adherence and cancer-related outcomes, including cancer incidence and survival [4–6]. Most studies have focused on specific cancer sites, mainly breast and colorectal cancers [4, 7–12].

In the UK Biobank prospective cohort study, which recruited >500,000 participants across the UK, greater adherence to the WCRF/AICR Recommendations (i.e., higher 2018 WCRF/AICR Scores) was associated with a lower relative risk for cancer overall and for individual cancer sites including breast and colorectum [13]. Further, in the NIH-AARP Diet & Health Study, higher adherence scores were associated with lower risk of 17 cancer types (combined) among older adults, which have collectively been linked to body weight, physical activity, dietary intake, and alcohol consumption, as well as with risk of breast, colorectal, and lung cancer [9].

The aim of this study was to investigate associations between the 2018 WCRF/AICR Score and risk of i) any cancers, ii) the 17 WCRF/AICR-reviewed cancers (combined), and iii) sixteen individual cancer sites in a US population using data from the Prostate, Lung, Colorectal, and Ovarian (PLCO) Cancer Screening Trial. This is the first study to comprehensively assess associations between the score and 16 cancer sites individually, including some not investigated previously e.g., glioma and melanoma, as well as with all cancers combined and lifestyle-related cancers combined. Only one other study has explored associations between the 2018 WCRF/AICR Score and cancer incidence in PLCO, and this was specific to pancreatic cancer incidence [5].

## Methods

### Study design

The PLCO Cancer Screening Trial is a large randomized controlled trial to determine whether screening for prostate, lung, colorectal, and ovarian cancers would reduce cancer-specific mortality [14, 15]. The PLCO Cancer Screening Trial is registered with ClinicalTrials.gov: NCT00002540 (Prostate), NCT01696968 (Lung), NCT01696981 (Colorectal), NCT01696994 (Ovarian), and NCT00339495 (EEMS).

Approximately 155,000 males and females, aged 55 to 74 years, were recruited from ten PLCO Screening Centers across the U.S. between November 1993 and July 2001. Exclusion criteria included: being aged <55 or >74 years, having a history of prostate, lung, colorectal, or ovarian cancer; current treatment for cancer (except basal or squamous cell skin cancer); prior surgical removal of entire prostate, entire colon, one lung, or both ovaries; participating in other cancer screening or primary prevention trials. Participants were individually randomized to the control arm or intervention arm in equal proportions, and randomization was stratified by age and sex within each screening center. Participants randomized to the intervention arm were invited to receive screening exams for prostate, lung, colorectal and ovarian cancers, and those allocated to the control arm received usual care from their healthcare provider.

### Inclusion criteria for present analytic sample

**Fig. 1:**
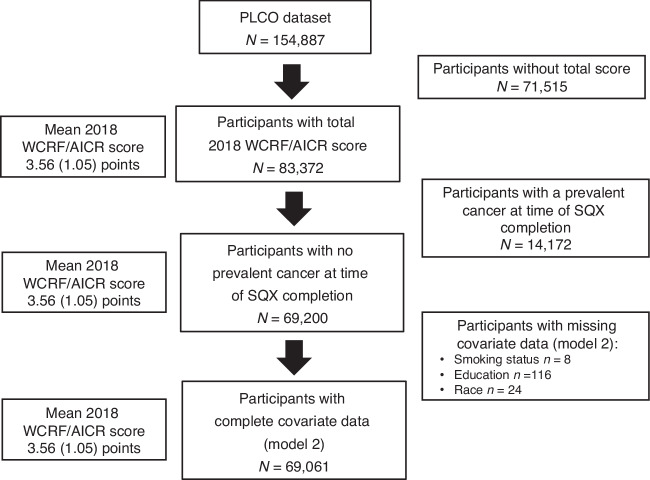
Flowchart illustrating analytical cohort from prostate, lung, colorectal, and ovarian cancer screening trial participants.

Participants were excluded from this analysis if it was not possible to calculate a total 2018 WCRF/AICR Score (i.e., they were missing data for any of the score components), if they were missing data for the main covariates included in the statistical model (Model 2), and if they had a prevalent cancer at time of completion of the Supplemental Questionnaire (SQX) (baseline for this study) (Fig. 1).

### Data collection

Participant information, including demographics, smoking history (status, years since quitting smoking, number of cigarettes smoked per day), anthropometrics (height, weight, BMI), medical history, and family history of cancer, was collected using the Baseline Questionnaire (BQ), which was completed by 96.8% of participants. A further SQX was introduced in 2006 to repeat assessment of some baseline factors, including demographics and smoking, and to collect new data on lifestyle factors, including physical activity and breastfeeding. All living and actively followed participants were mailed the SQX (134,848 participants; 87% of the 154,897 originally enrolled). Overall, 104,478 individuals responded to the one-time mailing (a response rate of 77.5%) [15].

BMI data were calculated using self-reported data on current weight and height collected in the SQX (if available), or BQ. Moderate-to-vigorous physical activity (MVPA) levels were assessed using physical activity data collected as part of the SQX, which asked “Over the last 12 months, on average, how many days per week did you spend in any moderate physical activity where you worked up a light sweat or increased your breathing and heart rate to moderately higher levels?“ for moderate activity, and “physical activity strenuous enough to work up a sweat or to increase your breathing and heart rate to very high levels?” for vigorous physical activity. For each activity, participants are also asked “Over the last 12 months, on average, how long was each session of moderate activity?” and “Over the last 12 months, on average, how long was each session of strenuous activity?”.

Dietary data were collected in both trial arms using the Diet History Questionnaire (DHQ), a food frequency questionnaire (FFQ) developed and validated by the National Cancer Institute that captures intake of 124 foods over past 12 months [16, 17]. In December 1998, five years into the study, the DHQ was administered to both arms of the trial such that 77% of all participants completed the DHQ. Participants randomized to the control arm of the trial prior to December 1998 were offered the DHQ in 1999 or 2000, and those randomized after introduction of the DHQ were administered the DHQ at baseline. Participants randomized to the intervention arm before December 1995 were offered the DHQ in 1998, and those after this date were given the DHQ around their third anniversary of randomization. Raw questionnaire responses were processed to derive dietary variables including food and nutrient intakes in grams or frequencies per day/week, calculated by the DietCalc software, which considers frequency, serving size, and other responses, in conjunction with national dietary data from USDA’s 1994-96 Continuing Survey of Food Intakes by Individuals (CSFII) [18]. A data collection timeline is presented in Supplementary Figure 1.

### 2018 WCRF/AICR Score

The 7-point version of the 2018 WCRF/AICR Score was operationalized as described by Shams-White and colleagues [2, 3] and adapted to the data available in PLCO (Table 1). Each component (i.e., recommendation) was weighted equally with evidence-based cut-points, as detailed in Table 1. Sub scores could range from 0 to 1 points, with a total score ranging 0–7 points (and a higher score indicating greater adherence to the recommendations). An 8-point version of the 2018 WCRF/AICR Score was also created, incorporating the optional eight score component regarding breastfeeding, for breast cancer-focused analyses.

**Table 1:** Operationalization of the 2018 WCRF/AICR Score in the Prostate, Lung, Colorectal, and Ovarian Cancer Screening Trial.

2018 WCRF/AICR Recommendation	Operationalization of Recommendations	Points
1. Be a healthy weight	BMI (kg/m^2^)	
	18.5–24.9	1
	25–29.9	0.5
	<18.5 or ≥30	0
2. Be physically active	Total moderate-vigorous physical activity (min/wk)	
	≥150	1
	75–150	0.5
	<75	0
3. Eat a diet rich in wholegrains, vegetables, fruit and beans	Fruits and vegetables (g/day)	
	≥400	0.5
	200– < 400	0.25
	<200	0
	Total fiber (g/day)	
	≥30	0.5
	15– < 30	0.25
	<15	0
4. Limit consumption of “fast foods” and other processed foods high in fat, starches or sugars	Percent of total kcal from ultra-processed foods (aUPFs)	
	Tertile 1 (lowest)	1
	Tertile 2	0.5
	Tertile 3 (highest)	0
5. Limit consumption of red and processed meat	Total red meat and processed meat (g/wk)	
	Red meat ≤500 and processed meat <21	1
	Red meat ≤500 and processed meat 21– <100	0.5
	Red meat >500 or processed meat ≥100	0
6. Limit consumption of sugar-sweetened drinks	Total sugar-sweetened drinks (g/day):	
	0	1
	>0– ≤ 250	0.5
	>250	0
7. Limit alcohol consumption	Ethanol intake (g/day)	
	0	1
	>0– ≤28 (2 drinks) males and ≤14 (1 drink) females	0.5
	>28 (2 drinks) males and >14 (1 drink) females	0
8. For mothers, breastfeed your baby if you can (optional)	Cumulative duration of exclusive breastfeeding (months)	
	≥6 months	1
	0–<6months	0.5
	Never	0

To assess adherence to the recommendation to ‘Be a healthy weight’, only data on BMI were available and not on the subcomponent regarding waist circumference. Therefore, in line with guidance on the standardized score, the BMI subcomponent values were doubled to retain the 0–1 point range [2, 3].

Adherence to the recommendation to ‘Be physically active’ was assessed using responses to the aforementioned SQX questions. Minutes of moderate and vigorous physical activity per week were calculated by multiplying the responses to the frequency questions by the duration of each activity session.

Adherence to the remaining five components related to diet and alcohol was assessed using data collected from the DHQ. The recommendation to ‘eat a diet rich in wholegrains, vegetables, fruits and beans’ comprised two sub-recommendations regarding the intakes of i) fruits and vegetables and ii) dietary fiber. Adherence to the fruits and vegetables intake sub-component was assessed by calculating the total intake of fruits and vegetables in grams per day.

DHQ food items were individually classified according to the Nova classification system to identify ultra-processed foods (UPFs) (please see Supplementary Methods). Only UPFs not already contributing to other score components, excluding items such as sugar-sweetened drinks and processed meats, were included in the ‘adapted UPFs’ (aUPFs) component of the score. The percentage of total calories from aUPFs was calculated for each participant. Points were allocated by categorizing participants into tertiles of total energy intake from aUPFs; those in the highest tertile scored 0 points, in the middle tertile were given 0.5 points, and in the lowest tertile were given 1 point.

To assess adherence to the recommendation pertaining to the intake of red and processed meats, intakes of red meats (excluding processed meats) based on responses to multiple items (veal, venison, lamb, steak, beef, hamburger, meatloaf, pork, short ribs, liver, as well as disaggregated from pasta meat sauces, lasagna, ravioli, etc., pizza, and chilli), and processed meats (ham, bacon, sausage, hot dogs, and cold cuts) were calculated in grams per week.

Consumption of sugar-sweetened drinks was calculated from data on the intake of regular soda and regular fruit drinks (such as cranberry cocktail, Hi-C, lemonade, or Kool-Aid), and converted to grams per day.

Adherence to the recommendation regarding alcohol consumption was assessed by calculating total alcohol (ethanol) intake in grams per day based on the intake of alcohol from beer, wine, and liquor.

For the 8-point version of the 2018 WCRF/AICR Score, responses to the question on total breastfeeding duration (in months) in a lifetime, collected using the SQX, were used.

### Outcome ascertainment

Incident cancer cases were identified either with linkage to population-based cancer registries or by self-report supplemented by linkage to the National Death Index and medical record review [14]. Participants were followed from SQX assessment until date of first primary cancer diagnosis, death, withdrawal from the study, or the end of follow-up (December 31, 2017), whichever came first. For site-specific cancer analyses, other first primary cancers were censored at diagnosis date. Information on cancer site, histology and stage was collected. Because individual studies used different versions of the International Classification of Diseases for Oncology (ICD-O) to classify cancers, our study coded all cancers according to the SEER Program Incidence Site Recode [19] based on ICD-*O*-3 [20]. In this analysis, we considered incident first primary diagnoses of any cancers and any of the 17 cancer sites highlighted in the 2018 WCRF/AICR Third Expert Report where evidence suggests causal links to body weight, physical activity, dietary intake, and alcohol consumption, including mouth, pharynx, and larynx ICD-O-3 site (C000-009, C0019-029, C079-C089, C040-C049, C030-C039, C050-C059, C060-C069, C090-C099, C100-C109, C129, C130-C139, C140, C142-C148), nasopharynx (C110-C119), esophageal (C150-C159), lung (C340-C349), stomach (C160-C169), pancreatic (C250-C259), gallbladder (C239), liver (C220), colorectal (C180-189, C199, C209, C260), breast (C500-C509), ovarian (C569), endometrial (C540-C549), cervical (C530-C539), prostate (C619), kidney (C649, C659), bladder (C670-C679), and melanoma (C440-C449) [1], as well as individual cancer sites with ≥100 incident cases i.e., prostate, breast, hematopoietic, lung, melanoma, bladder, colorectal, pancreatic, renal, endometrial, upper GI, head and neck, ovarian, glioma and thyroid, plus liver cancer (96 cases), ascertained during the follow-up period [21].

### Statistical analyses

Participant characteristics were tabulated overall and according to study-specific score tertiles. Differences according to score tertiles were assessed using χ2 and Kruskal-Wallis tests for categorical and continuous variables, respectively.

Associations between 2018 WCRF/AICR Score and cancer incidence were tested using Cox proportional hazard regression models to estimate hazard ratios (HRs) and 95% confidence intervals (95% CIs). Risk estimates and corresponding 95% CIs were estimated per 1-point increment in score (continuous), as well as according to study-specific tertiles, with the lowest tertile (lowest adherence) as the reference group. All models used person-years on study as the underlying time scale, and participants with less than 1 year follow-up time (difference between the age at first cancer diagnosis or trial exit and the age at exposure i.e., SQX completion) were excluded.

We ran an unadjusted model, Model 1 adjusted for age and sex (if applicable), and Model 2 additionally adjusted for trial arm, race, education, smoking status (never, former, current), cigarette smoking (derived from data on smoking status, time since quitting, and number of cigarettes smoked per day), and cigar or pipe smoking. For female cancers (i.e., breast, ovarian, and endometrial), an additional model (Model 3) was run further adjusting for female-specific factors (i.e., contraceptive use, parity, female hormone use) and family history of that cancer. Pre-defined sub-group analyses were stratified according to sex and smoking status (never, current, former). We additionally modelled the joint effects of 2018 WCRF/AICR Score and smoking status by creating a combined variable, using never smokers in the highest score tertile as the reference group. Statistical analyses were conducted using STATA v18.5 (StataCorp, College Station, TX). *P* values < 0.05 were considered statistically significant.

## Results

### Participant characteristics

After applying the study exclusion criteria, a total of 69,061 PLCO Trial participants were included in the present analysis (Fig. 1), and their characteristics (overall and according to score tertiles) are presented in Table 2. The mean age at enrollment was 62 (range 55–74), 54% of participants were female, and 92% were non-Hispanic White. Participants in the highest 2018 WCRF/AICR Score tertile were more likely to be older, female, Asian, college graduates, and never smokers.

**Table 2:** Prostate, Lung, Colorectal, and Ovarian Cancer Screening Trial participant characteristics according to approximate 2018 WCRF/AICR Score tertiles of the study population.

	All	Tertile 1 (0–3 points)	Tertile 2 (3.25–4 points)	Tertile 3 (4.25–7 points)
Number (*n*)	69,061 (100)	23,569 (34.1)	24,216 (35.1)	21,276 (30.8)
Age (years)	61.9 (5.1)	61.5 (5.1)	62.0 (5.1)	62.3 (5.2)
Females	36,995 (53.6)	10,774 (45.7)	12,578 (52.0)	13,643 (64.1)
Trial arm				
*Intervention*	35,893 (52.0)	12,285 (52.1)	12,536 (51.8)	11,072 (52.0)
*Control*	33,168 (48.0)	11,284 (47.9)	11,680 (48.2)	10,204 (48.0)
Race				
*Non-Hispanic White*	63,611 (92.1)	21,806 (92.5)	22,349 (92.3)	19,456 (91.5)
*Non-Hispanic Black*	1717 (2.5)	706 (3.0)	553 (2.3)	458 (2.2)
*Hispanic*	979 (1.4)	304 (1.3)	358 (1.5)	317 (1.5)
*Asian*	2341 (3.4)	585 (2.5)	824 (3.4)	932 (4.4)
*Pacific Islander*	285 (0.4)	119 (0.5)	91 (0.4)	75 (0.4)
*American Indian*	128 (0.2)	49 (0.2)	41 (0.2)	38 (0.2)
Education				
*LT Highschool*	3300 (4.8)	1428 (6.1)	1153 (4.8)	719 (3.4)
*Highschool graduate*	15,764 (22.8)	6405 (27.2)	5430 (22.4)	3929 (18.5)
*Some college/ post-high school training*	23,763 (34.4)	8482 (36.0)	8298 (34.3)	6983 (32.8)
*College graduate +*	26,234 (38.0)	7254 (30.8)	9335 (38.6)	9645 (45.3)
Smoking status				
*Never*	34,046 (49.3)	10,178 (43.2)	11,904 (49.2)	11,964 (56.2)
*Current*	5548 (8.0)	2801 (11.9)	1815 (7.5)	932 (4.4)
*Former*	29,467 (42.7)	10,590 (44.9)	10,497 (43.4)	8380 (39.4)
Total 2018 WCRF/AICR Score (points)	3.6 (1.0)	2.4 (0.5)	3.6 (0.3)	4.8 (0.5)

Data are presented as means and standard deviation (SD) for continuous variables, and as number and percentage (%) of participants for categorical variables. Percentage of participants represents the value within that column (i.e., of all participants and within each tertile), with the exception of the row ‘Number of participants’ where the (%) value represents the percentage of all of the participants in each tertile (i.e., within that row).

Comparisons between the characteristics of all PLCO Trial participants and those included in the present study are presented in Supplementary Table 1. Included participants were similar with respect to age, the proportion of males and females, and mean BMI; however, they comprised a greater proportion of Non-Hispanic White participants and participants educated to at least College level.

### Adherence to the 2018 WCRF/AICR cancer prevention recommendations in the PLCO cohort

The mean 2018 WCRF/AICR Score across all participants was 3.6 points (range 0–7; Table 2). When adding the eighth optional component regarding breastfeeding to the score for female participants (score range 0–8 points), the mean score was 4.2 points (SD 1.2).

**Fig. 2:**
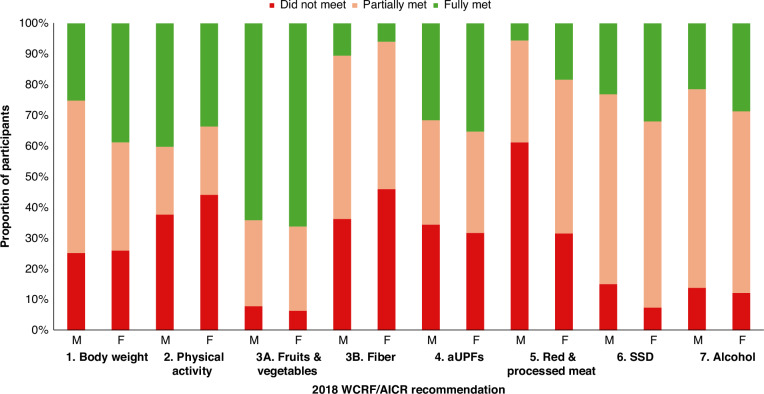
Adherence to the 2018 WCRF/AICR Score components for male and female prostate, lung, colorectal, and ovarian cancer screening trial participants. (*n* = 69,061 total, *n* = 32,066 males, *n* = 36,995 females). Abbreviations: aUPF adapted ultra-processed foods, F female, M male, SSD sugar-sweetened drinks.

The proportions of male and female participants not meeting, partially meeting, and fully meeting each of the seven score components are presented in Fig. 2. The recommendations most adhered to by PLCO participants were those regarding body weight, physical activity, and the sub-recommendation on intake of fruits and vegetables. Participants were less likely to meet the recommendations on the intake of red and processed meats and the sub-recommendation regarding dietary fiber intake. Adherence to the recommendations were similar for males and females, with the exception of the recommendations on red and processed meat (61% males vs. 31% females did not meet), sugar-sweetened drinks (15% males vs. 7% females did not meet), and the sub-recommendation on dietary fiber intake (11% males vs. 6% females fully met).

### 2018 WCRF/AICR Score and risk of cancer overall

During a median follow-up time of 10 years, 11,109 participants developed cancer. The most common cancers were prostate (19% of cases), breast (15% of cases), hematopoietic (12% of cases), and lung (11% of cases).

**Fig. 3:**
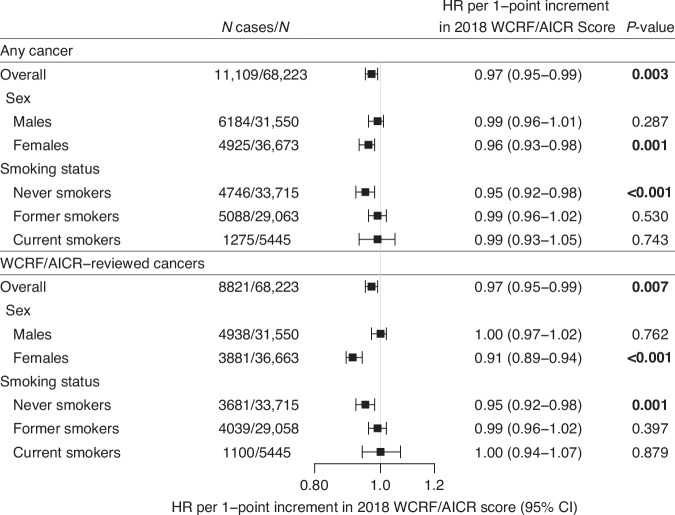
Associations between 2018 WCRF/AICR Score and risk of all cancers combined and of WCRF/AICR-reviewed cancers combined in the prostate, lung, colorectal, and ovarian cancer screening trial. Data are presented as hazard ratios (HR) and corresponding 95% confidence intervals (95% CIs) per 1-point increment in 2018 WCRF/AICR Score. Analyses were performed overall as well as stratified according to sex and to smoking status at baseline (never, former, current smokers). Model 1 adjusted for age, sex (if applicable). Model 2 adjusted for age, sex (if applicable), trial arm, race, education, smoking status, cigarette, cigar or pipe smoking.

The results for the associations between score and risk of any cancer (cancer overall) are presented in Fig. 3, Supplementary Table 2 and Supplementary Table 3. When the score was assessed continuously, there was a 3% reduction in the risk of developing any cancer per 1-point increment in score (HR: 0.97; 95% CI: 0.95–0.99; *p* = 0.003). Stratified analyses revealed that this association was observed for female (HR: 0.96; 95% CI: 0.93–0.98; *p* = 0.001) but not male (HR: 0.99; 95% CI: 0.96–1.01; *p* = 0.287) participants, and only significant in never smokers. Associations appeared stronger in females than males and in never smokers; however, there was no statistical evidence for effect measure modification by sex (p-heterogeneity=0.136) or smoking status (p-heterogeneity=0.092), which may be due to limited statistical power.

Participants in the highest score tertile (scoring ≥4.25 points), had a 6% lower risk of developing any cancer compared with those in the lowest score tertile, scoring ≤3 points (HR_T3 vs. T1_: 0.94; 95% CI 0.89–0.99; p_trend_ = 0.016; Supplementary Table 3). Following stratification according to smoking status, a lower relative risk of any cancer was observed in never smokers (HR_T3 vs. T1_: 0.89; 95% CI: 0.83-0.96; p_trend_ = 0.002), but not in current or former smokers. When these analyses were stratified according to sex and smoking status, a lower relative risk of any cancer was again observed only in female never smokers (HR_T3 vs. T1_: 0.85; 95% CI: 0.77–0.94; p_trend_ = 0.001) but not in former or current smokers, or in males. When 2018 WCRF/AICR Score and smoking status were jointly modelled, participants in the lowest score tertile and who were current smokers had the highest risk compared with never smokers in the highest score tertile (Supplementary Fig. 2).

### 2018 WCRF/AICR Score and risk of 17 cancer sites reviewed by the WCRF/AICR

Overall, each 1-point increment in 2018 WCRF/AICR Score was associated with a 3% reduction in the risk of these 17 cancer sites combined (HR: 0.97; 95% CI: 0.95–0.99; *p* = 0.007). and this association was again stronger in never smokers (HR: 0.95; 95% CI: 0.92–0.98; *p* < 0.001; Fig. 3 and Supplementary Table 4). After stratifying according to sex, inverse associations remained in females (HR: 0.91; 95% CI: 0.89–0.94; *p* < 0.001), namely those who never smoked (HR: 0.92; 95% CI: 0.88–0.96; *p* < 0.001), but not males (HR: 1.00; 95% CI: 0.97–1.02; *p* = 0.762).

Similar findings were observed when analyzing differences in cancer risk between 2018 WCRF/AICR Score tertiles (Supplementary Table 5); participants in the highest tertile (scoring ≥4.25 points) had a 6% lower risk of developing the 17 cancer sites reviewed by the WCRF/AICR (HR_T3 vs. T1_: 0.94; 95% CI: 0.89–0.99; *p* = 0.048). After stratifying according to smoking status, this risk reduction was stronger and only significant in participants who had never smoked (HR_T3 vs. T1_: 0.90; 95% CI: 0.83–0.98; *p* = 0.009).

### 2018 WCRF/AICR Score and risk of individual cancer sites

**Fig. 4:**
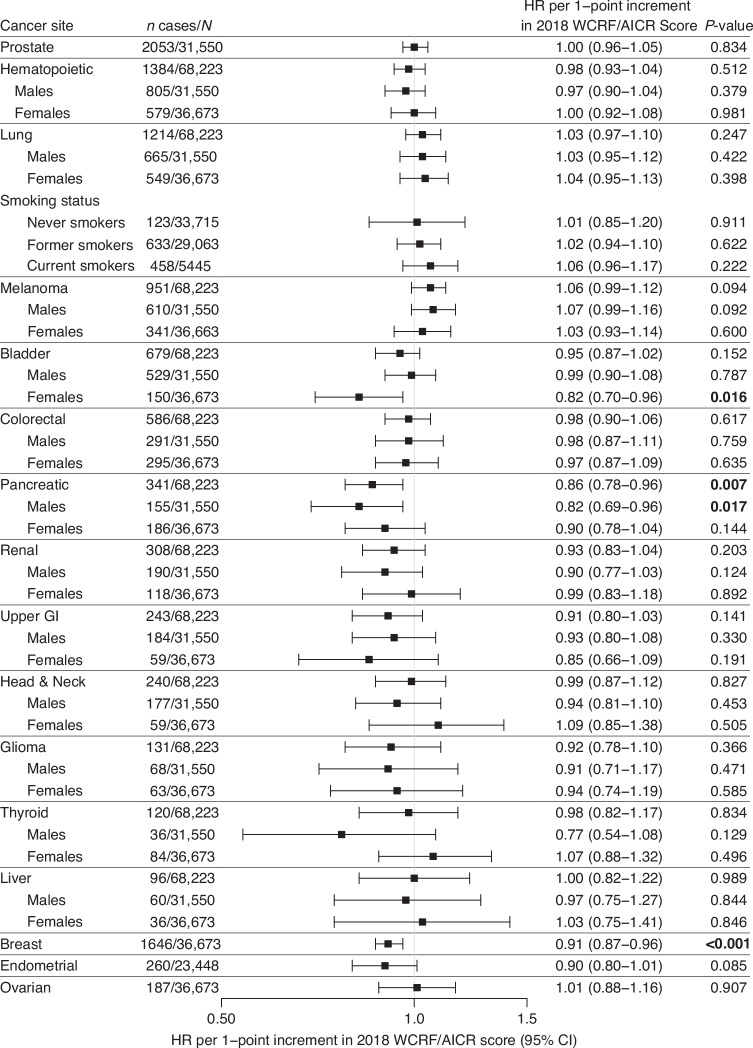
Associations between 2018 WCRF/AICR Score and risk of individual cancer sites in the prostate, lung, colorectal, and ovarian cancer screening trial. Data are presented as hazard ratios (HR) and corresponding 95% confidence intervals (95% CIs) per 1-point increment in 2018 WCRF/AICR Score. Analyses were performed overall as well as stratified according to sex and to smoking status at baseline (never, former, current smokers). Model 1 adjusted for age, sex (if applicable). Model 2 adjusted for age, sex (if applicable), trial arm, race, education, smoking status, cigarette, cigar or pipe smoking.

Overall, a higher 2018 WCRF/AICR Score was associated with lower risk of pancreatic cancer (HR: 0.86; 95% CI: 0.78–0.96; *p* = 0.007), particularly for male participants (HR: 0.82; 95% CI: 0.69–0.96; *p* = 0.017; Fig. 4 and Supplementary Table 6). An inverse association was also observed among females, albeit not statistically significant (HR: 0.90; 95% CI: 0.78–1.04; *p* = 0.144). Each 1-point increment in Score was associated with an 18% lower relative risk of bladder cancer in females (HR per 1-point increment: 0.82; 95% CI: 0.70–0.96; *p* = 0.016) but not in males (HR: 0.99; 95% CI: 0.90–1.08; *p* = 0.787).

For female-specific cancers, the 2018 WCRF/AICR Score was inversely associated with breast cancer (HR per 1-point increment: 0.91; 95% CI: 0.87–0.96; *p* < 0.001) and endometrial cancer risk (HR per 1-point increment: 0.90; 95% CI: 0.80–1.01; *p* = 0.085). Additional adjustment for female-specific risk factors in model 3 (contraceptive use, parity, female hormone use) and family history of breast (or endometrial) cancer did not attenuate the associations for breast (HR per 1-point increment: 0.91; 95% CI: 0.87–0.96; *p* < 0.001) or endometrial cancer (HR per 1-point increment: 0.90; 95% CI: 0.80–1.01; *p* = 0.078). Females in the highest score tertile scoring ≥4.25 points had a 19% lower risk of developing breast cancer compared with those in the lowest tertile scoring ≤3 points (HR_T3 vs. T1_: 0.81; 95% CI: 0.72–0.92; p_trend_ = 0.001; Supplementary Table 7). When investigating associations with the 8-point version of the 2018 WCRF/AICR Score, which includes the optional eighth score component regarding breastfeeding, a similar inverse association was observed with higher scores and breast cancer incidence (HR per 1-point increment: 0.94; 95% CI: 0.90–0.98; *p* = 0.002; Supplementary Table 6; and HR_T3 vs. T1_: 0.85; 95% CI: 0.75–0.96; p_trend_ = 0.010, Supplementary Table 7).

Lastly, for prostate, lung, colorectal, and ovarian cancer incidence, analyses were repeated stratified according to trial arm. With the exception of ovarian cancer (p_heterogeneity_= 0.020), there was no significant interaction between 2018 WCRF/AICR Score and trial arm (Supplementary Table 8).

## Discussion

Our study investigated whether adherence to the 2018 WCRF/AICR Cancer Prevention Recommendations, assessed using the standardized 2018 WCRF/AICR Score, was associated with the risk of cancers in the PLCO cohort. To our knowledge, only one study has explored associations between adherence to the 2018 Cancer Prevention Recommendations and cancer in the PLCO trial. Zhang and colleagues fully operationalized the 2018 WCRF/AICR Score, with the exception of the sub-component regarding waist circumference as these data were not collected, and investigated associations with pancreatic cancer incidence and mortality [5].

Overall, over a median follow-up time of 10 years, we observed a 3% lower relative risk of any cancer per 1-point increment in the 2018 WCRF/AICR Score. The observed associations were stronger in females and in participants who had never smoked. We also found a 3% lower relative risk per 1-point increment in score of any of the 17 lifestyle-associated cancers (combined) reviewed by the WCRF and AICR, including mouth, pharynx, and larynx, nasopharynx esophageal, lung, stomach, pancreatic, gallbladder, liver, colorectal, breast, ovarian, endometrial, cervical, prostate, kidney, bladder, and melanoma. Similar to our results for cancer overall, we found stronger inverse associations in never smokers (5% lower relative risk per 1-point increment) and females (9% lower relative risk per 1-point increment). These findings are comparable to those reported by Korn and colleagues, who found a 6% and 9% lower relative risk for these 17 cancer sites (combined) per 1-point increment in score in the NIH-AARP Diet and Health Study in males and females, respectively, who had never smoked [9]. However, in the present study, findings were only significant in females, not males; this is likely driven by the significant associations observed for breast cancer incidence, compared with other cancers. Further, in contrast to our study, Korn and colleagues also observed inverse associations among former smokers, which may be explained, at least in part, by the larger sample size and longer follow-up for cancer incidence in NIH-AARP [9]. The observed stronger associations in non-smokers for cancer overall and for the 17 cancer sites reviewed by the WCRF/AICR may be due to the potent carcinogenic effects of smoking through pathways such as inducing DNA damage and oxidative stress, diminishing protective effects of healthier lifestyle behaviors. Further, there is possible residual confounding and additional adverse behaviors among smokers that could attenuate associations between lifestyle and cancer risk. It is also important to note that the types of cancers will vary by smoking status, and lifestyle factors may not play an important role in risk of smoking-driven cancers such as lung cancer.

A strength of this study is that we additionally estimated associations between the score and individual cancer types, defined according to primary site and histology data fields submitted to SEER by the cancer registries [19]. In these analyses, we observed a 14% lower risk of pancreatic cancer per 1-point increment in score. Additionally, the relative risk of developing pancreatic cancer was 30% lower in individuals scoring ≥4.25 points compared with those scoring ≤3 points. Although our follow-up period was longer, these findings agree with those from Zhang et al., who reported a 12% lower relative risk of pancreatic cancer per 1-point increment in 2018 WCRF/AICR Score in PLCO over a mean follow-up of 8.9 years [5]. Further, a 13% lower relative risk of pancreatic cancer per 1-point increment in 2018 WCRF/AICR Score was reported in a UK cohort, which was substantially attenuated after adjusting for smoking status [13].

Higher adherence to the recommendations was also associated with lower relative risk of female breast cancer, with a 19% lower relative risk in females in the highest compared with the lowest score tertile. To date, breast cancer incidence is the most widely studied cancer with respect to the 2018 WCRF/AICR Score, and studies have consistently reported an inverse association [4]. In a systematic review and meta-analysis including seven studies of breast cancer incidence, including both pre- and post-menopausal disease, each 1-point increment in adherence score was associated with an 11% lower relative risk of breast cancer [4]. In the US NIH-AARP Diet and Health Study, researchers found a 7%, 9% and 19% lower relative risk of breast cancer per 1-point increment in score in never, former, and current smokers, respectively [9]. In the UK Biobank prospective cohort study, a 10% reduction in the risk of breast cancer per 1-unit increment in 2018 WCRF/AICR Score has also been reported [13]. In a case-control study conducted in South Africa, which also fully operationalised the 2018 WCRF/AICR Score and additionally included the eighth component regarding breastfeeding, there was a 14% lower breast cancer risk per 1-point increment in score [10]. In contrast, non-significant inverse associations between score and overall breast cancer risk and breast cancer *in situ* have been reported in a Spanish prospective cohort study of 10,930 women [7] and in 232,848 women from the UK Biobank [8], respectively. Potential mechanisms for the protective effects of a healthier lifestyle, as indicated by a higher 2018 WCRF/AICR Score, on breast cancer risk include reduced inflammation, lower oxidative stress, favorable hormonal changes, and improved insulin sensitivity.

Additionally, among females, each 1-point increment in score was associated with an 18% reduction in the relative risk of bladder cancer. Associations between adherence to the Cancer Prevention Recommendations and the incidence of bladder cancer have not been reported previously. However, the WCRF/AICR concluded that there is limited suggestive evidence that greater consumption of fruits and vegetables decreases the risk of bladder cancer [1]. Potential mechanisms for the protective effects of fruits and vegetables, and other healthy lifestyle components of the Cancer Prevention Recommendations, include a reduction in DNA damage and oxidative stress, attenuation of inflammation, and improved insulin sensitivity, as well as reduced exposure to urinary carcinogens through compounds present in cruciferous vegetables [22].

Lastly, we observed a trend for a 10% lower relative risk of endometrial cancer per 1-point increment in score. In a case-control study conducted in Italy, Esposito et al. reported a 28% lower relative risk of endometrial cancer per 1-point increment in score [23]. The WCRF/AICR have judged that there is strong evidence for physical activity probably reducing endometrial cancer risk, and strong evidence for body fatness increasing endometrial cancer risk, which are two lifestyle components of the 2018 WCRF/AICR Score [1]. Physical activity may protect against endometrial cancer by reducing estradiol and increasing sex hormone binding globulin levels, partly via promoting a healthy body weight [1]. In contrast, greater levels of body fatness are associated with hormonal imbalances, including increased levels of estrogens, insulin resistance, and chronic inflammation, which are associated with risk of endometrial, as well as other, cancers [1].

In contrast to other studies [4], we did not observe associations between adhering to the 2018 WCRF/AICR Cancer Prevention Recommendations and colorectal cancer incidence in the PLCO Cancer Screening Trial. For example, in two prospective cohorts of women and men (Nurses’ Health Study (NHS) and the Health Professionals Follow-up Study (HPFS), respectively), a 22% lower relative risk of colorectal cancer per 1-point increment in adherence score was observed in men but not in women [11]. In another US cohort, Korn and colleagues reported a 10-14% lower relative risk of colorectal cancer among male and female never and former smokers [9]. We also did not observe associations with kidney, esophageal, ovarian, liver, and lung cancer risk, which have been reported previously [9, 13, 24]. Observed differences may be explained in part by the smaller number of incident colorectal cancer cases in our analytic sample or by the unique design of our study, which entailed colorectal cancer screening via flexible sigmoidoscopy, with referral for medical intervention for those who screened positive, in the screening arm of the PLCO Trial.

### Strengths and limitations

This study used data from a large and geographically diverse US cohort. However, our participants were older and mostly Non-Hispanic White, potentially limiting the generalizability of our results. A strength of this study is that in addition to combined cancer outcomes, we explored associations between the 2018 WCRF/AICR Score and risk of common cancer types, defined according to SEER and based on primary site and histology data. Thus, our findings provide new data on cancers, such as glioma and thyroid cancers, that are not well studied previously with respect to lifestyle recommendations for cancer prevention. Our results should be interpreted with caution, we did not choose *a priori* to adjust for multiple testing.

A further strength of this study is that we operationalized seven components of the 2018 WCRF/AICR standardized scoring system. Although we were not able to assess the waist circumference sub-component of the ‘be a healthy body weight’ recommendation, as advised by the score creators, we doubled the sub-score for BMI to retain the 0-1 point total range for that component [2, 3]. We examined the score on a continuous scale, allowing for comparability with other studies. Further, for breast cancer, we operationalized an 8-point version of the 2018 WCRF/AICR Score which included breastfeeding. Similar attenuation of associations between score and breast cancer risk with the addition of breastfeeding have been observed by Barrios-Rodríguez et al. [7].

However, there are several limitations associated with the data used to calculate the score; for example, the data were only collected at one timepoint, were self-reported, and the data collection tools may be prone to measurement error (e.g., misreporting of dietary intakes). A limitation of our study is that data used to calculate the score were not collected simultaneously; for example, BMI data were collected as part of the BQ and dietary data were collected when the DHQ was introduced five years into the trial. Nonetheless, evidence exists to suggest that dietary intake patterns are relatively stable over time, particularly in middle-aged adults [25, 26]. We excluded participants with missing data for any of the 2018 WCRF/AICR Score components and for the major confounders (e.g., smoking) included in our model. Nonetheless, the included participants were comparable in terms of characteristics to the full PLCO cohort. Although we adjusted for established cancer risk factors using detailed data collected prior to first cancer diagnosis, still residual confounding by unknown or unmeasured confounders is possible. Further, it must be noted that participants in a cancer screening trial may exhibit differential behavior, including potentially adopting healthier lifestyles, compared with the general population. Although participants in the control and intervention groups were comparable at study baseline, there are additional possible behavioral differences. Further, it is possible that our null findings for prostate, lung, colorectal, and ovarian cancers may reflect the screening component for these cancers in the intervention arm, which may have attenuated associations with lifestyle or led to changes in behavior during follow-up. It is also possible that those randomized to the control group were more adherent to screening guidelines given their willingness to enroll in the study. Prior PLCO studies have reported that the PLCO cohort is healthier and more well educated than the general US population. However, when we repeated analyses stratified according to trial arm, there was no significant interaction between 2018 WCRF/AICR Score and trial arm, except for ovarian cancer.

## Conclusions

In conclusion, our findings support adherence to the 2018 WCRF/AICR Cancer Prevention Recommendations for reducing risk of developing any cancer as well as specific cancers, including female breast and pancreatic cancer, in a US cohort. Although the 3% lower risk per 1-point increment in 2018 WCRF/AICR Score, equating to going from not meeting to fully meeting one recommendation or to increasing adherence to partially meeting from not meeting or to fully meeting from partially meeting two recommendations, is modest, the cumulative differences across the full range of up to 7 (or 8) points may translate to an impactful reduction in cancer risk. Further, given that lifestyle modifications are safe and low-cost, adopting healthier lifestyles may elicit meaningful effects at the population level.

## Supplementary information

Supplementary information

## Data Availability

Data are maintained by the National Cancer Institute, Division of Cancer Epidemiology and Genetics and Division of Cancer Prevention, and are available to bona fide researchers upon submission and approval of a research proposal, and subsequent completion of a Data Transfer Agreement.
