# Supplementary material for: Adherence to the 2018 World Cancer Research Fund/American Institute for Cancer Research Cancer Prevention Recommendations and risk of lifestyle-related cancers in the prostate, lung, colorectal, and ovarian cancer screening trial
Source: BJC Rep. 2025 Nov 19;3:81. doi: 10.1038/s44276-025-00195-6 (PMC12630754; doi:10.1038/s44276-025-00195-6)
Supplement: Supplementary file 1 — Supplementary information [file 44276_2025_195_MOESM1_ESM.docx]

**SUPPLEMENTARY MATERIAL**

**Supplementary Methods**

**Assessment of adapted Ultra-Processed Foods (aUPFs)**

Adapted Ultra-Processed Foods (aUPFs) in PLCO were assessed using an expert consensus method. Individual line items from the DHQ were categorized according to the Nova classification and only items in group 4 (UPFs) and that were not already accounted for in other score components were included (i.e., sugar-sweetened drinks, processed meats, and alcohol were excluded). Calories were calculated from the DHQ responses using the DietCalc software, which takes into account frequency and serving size, as well as additional aspects e.g., whether a soft drink is regular, diet, or sugar free. The nutrient database is based on national dietary data: USDA's 1994-96 Continuing Survey of Food Intakes by Individuals (CSFII) and the Nutrition Data Systems for Research (NDS-R). The percentage of calories from aUPFs consumed by each participant was calculated as a percentage of total calorie intake.

**Supplementary Tables**

**Supplementary Table 1 Characteristics of participants included and all Prostate, Lung, Colorectal, and Ovarian Cancer Screening Trial participants**

|  | **All PLCO participants** | **Included in complete case analysis** |
| --- | --- | --- |
| Number (n) | 154,887 | 69,061 |
| Age (years) | 62.6 (5.4) | 61.9 (5.1) |
| Females | 78,209 (50.5) | 36,995 (53.6) |
| Trial arm |  |  |
| *Intervention* | 77,443 (50.0) | 35,893 (52.0) |
| *Control* | 77,444 (50.0) | 33,168 (48.0) |
| Race |  |  |
| *Non-Hispanic White* | 132,572 (85.6) | 63,611 (92.1) |
| *Non-Hispanic Black* | 7,708 (5.0) | 1,717 (2.5) |
| *Hispanic* | 2,818 (1.8) | 979 (1.4) |
| *Asian* | 5,575 (3.6) | 2,341 (3.4) |
| *Pacific Islander* | 835 (0.5) | 285 (0.4) |
| *American Indian* | 389 (0.3) | 128 (0.2) |
| *Missing* | 4,990 (3.2) | 0 (0.0) |
| Education |  |  |
| *LT Highschool* | 11,080 (7.2) | 3,300 (4.8) |
| *Highschool graduate* | 34,394 (22.2) | 15,764 (22.8) |
| *Some college/ post-high school training* | 51,516 (33.3) | 23,763 (34.4) |
| *College graduate* + | 52,569 (33.9) | 26,234 (38.0) |
| *Missing* | 5,328 (3.4) | 0 (0.0) |
| Smoking status |  |  |
| *Never* | 69,268 (44.7) | 34,046 (49.3) |
| *Current* | 16,053 (10.4) | 5,548 (8.0) |
| *Former* | 64,615 (41.7) | 29,467 (42.7) |
| *Missing* | 4,951 (3.2) | 0 (0.0) |
| BMI (kg/m^2^)^a^ | 27.4 (5.1) | 27.4 (5.0) |

^a^BMI data missing for n=4,035 (2.6%) of participants.

**Supplementary Table 2 Associations between 2018 WCRF/AICR Score and risk of any cancer (all cancers combined) per 1-point increment in score, stratified by sex and smoking status, in the Prostate, Lung, Colorectal, and Ovarian Cancer Screening Trial**

| **All**  *11,109/68,223* |  |  | **Males**  *6,184/31,550* |  | **Females**  *4,925/36,673* |  |
| --- | --- | --- | --- | --- | --- | --- |
|  | **HR (95% CI)** | **P-value** | **HR (95% CI)** | **P-value** | **HR (95% CI)** | **P-value** |
| Unadjusted | **0.92 (0.90; 0.94)** | **<0.001** | **0.96 (0.94; 0.99)** | **0.002** | **0.94 (0.91; 0.96)** | **<0.001** |
| Model 1 | **0.94 (0.92; 0.96)** | **<0.001** | **0.95 (0.93; 0.97)** | **<0.001** | **0.93 (0.91; 0.96)** | **<0.001** |
| Model 2 | **0.97 (0.95; 0.99)** | **0.003** | 0.99 (0.96; 1.01) | 0.287 | **0.96 (0.93; 0.98)** | **0.001** |
| **Never smoker**  *4,746/33,715* |  |  | *2,152/12,367* |  | *2,592/21,339* |  |
| Unadjusted | **0.94 (0.91; 0.96)** | **<0.001** | 0.99 (0.95; 1.03) | 0.491 | **0.94 (0.91; 0.98)** | **0.002** |
| Model 1 | **0.95 (0.93; 0.98)** | **0.001** | 0.97 (0.93; 1.02) | 0.223 | **0.94 (0.90; 0.97)** | **<0.001** |
| Model 2 | **0.95 (0.92; 0.98)** | **<0.001** | 0.97 (0.93; 1.02) | 0.217 | **0.93 (0.90; 0.97)** | **<0.001** |
| **Current smoker**  *1,275/5,445* |  |  | *740/2,707* |  | *535/2,738* |  |
| Unadjusted | 0.95 (0.89; 1.00) | 0.051 | 0.99 (0.92; 1.08) | 0.876 | 0.96 (0.88; 1.05) | 0.377 |
| Model 1 | 0.96 (0.91; 1.02) | 0.227 | 0.97 (0.90; 1.05) | 0.511 | 0.95 (0.88; 1.04) | 0.281 |
| Model 2 | 0.99 (0.93; 1.05) | 0.743 | 1.00 (0.92; 1.09) | 0.953 | 0.98 (0.90; 1.06) | 0.579 |
| **Former smoker**  *5,088/29,063* |  |  | *3,292/16,476* |  | *1,796/12,587* |  |
| Unadjusted | **0.95 (0.92; 0.97)** | **<0.001** | 0.98 (0.95; 1.02) | 0.317 | 0.97 (0.93; 1.01) | 0.195 |
| Model 1 | **0.97 (0.94; 0.99)** | **0.019** | 0.97 (0.94; 1.00) | 0.088 | 0.96 (0.92; 1.01) | 0.106 |
| Model 2 | 0.99 (0.96; 1.02) | 0.530 | 0.99 (0.96; 1.03) | 0.647 | 0.99 (0.95; 1.04) | 0.641 |

Model 1 adjusted for age, sex (if applicable). Model 2 adjusted for age, sex (if applicable), trial arm, race, education, smoking status, cigarette, cigar or pipe smoking.

**Supplementary Table 3 Risk of any cancer (all cancers combined) according to 2018 WCRF/AICR Score tertiles, stratified by sex and smoking status, in the Prostate, Lung, Colorectal, and Ovarian Cancer Screening Trial**

|  | **All** | | | **Males** | | | **Females** | | |
| --- | --- | --- | --- | --- | --- | --- | --- | --- | --- |
|  | **Tertile 2**  **(3.25 – 4 points)** | **Tertile 3**  **(4.25 – 7 points)** | **P-trend** | **Tertile 2**  **(3.25 – 4 points)** | **Tertile 3**  **(4.25 – 7 points)** |  | **Tertile 2**  **(3.25 – 4 points)** | **Tertile 3**  **(4.25 – 7 points)** |  |
|  | **HR (95% CI)** | **HR (95% CI)** | **P trend** | **HR (95% CI)** | **HR (95% CI)** | **P trend** | **HR (95% CI)** | **HR (95% CI)** | **P trend** |
| **All**  *11,109/68,223* |  |  |  |  |  |  |  |  |  |
| Unadjusted | **0.95 (0.91; 0.99)** | **0.82 (0.78; 0.86)** | **<0.001** | 1.00 (0.94; 1.06) | **0.91 (0.85; 0.97)** | **0.008** | 0.95 (0.88; 1.01) | **0.86 (0.80; 0.92)** | **<0.001** |
| Model 1 | 0.97 (0.92; 1.00) | **0.87 (0.83; 0.91)** | **<0.001** | 0.98 (0.93; 1.04) | **0.88 (0.82; 0.94)** | **<0.001** | 0.94 (0.87; 1.00) | **0.85 (0.79; 0.91)** | **<0.001** |
| Model 2 | 1.00 (0.96; 1.05) | **0.94 (0.89; 0.99)** | **0.016** | 1.03 (0.97; 1.09) | 0.96 (0.90; 1.03) | 0.391 | 0.96 (0.90; 1.03) | **0.91 (0.85; 0.97)** | **0.007** |
| **Never smoker** | *4,746/33,715* |  |  |  |  |  |  |  |  |
| Unadjusted | 1.02 (0.95; 1.09) | **0.87 (0.81; 0.93)** | **<0.001** | 1.05 (0.95; 1.16) | 0.98 (0.88; 1.09) | 0.804 | 1.01 (0.92; 1.11) | **0.87 (0.79; 0.96)** | **0.003** |
| Model 1 | 1.02 (0.95; 1.09) | **0.90 (0.84; 0.97)** | **0.004** | 1.04 (0.94; 1.14) | 0.95 (0.86; 1.06) | 0.453 | 1.00 (0.91; 1.10) | **0.86 (0.78; 0.95)** | **0.001** |
| Model 2 | 1.02 (0.95; 1.09) | **0.89 (0.83; 0.96)** | **0.002** | 1.04 (0.94; 1.15) | 0.95 (0.85; 1.06) | 0.439 | 1.00 (0.90; 1.10) | **0.85 (0.77; 0.94)** | **0.001** |
| **Current smoker** | *1,275/5,445* |  |  |  |  |  |  |  |  |
| Unadjusted | 0.95 (0.84; 1.07) | 0.89 (0.76; 1.04) | 0.121 | 1.06 (0.90; 1.24) | 0.94 (0.74; 1.19) | 0.938 | 0.88 (0.72; 1.07) | 0.97 (0.78; 1.21) | 0.601 |
| Model 1 | 0.96 (0.85; 1.08) | 0.94 (0.80; 1.10) | 0.366 | 1.04 (0.88; 1.21) | 0.89 (0.70; 1.13) | 0.584 | 0.86 (0.70; 1.04) | 0.95 (0.76; 1.18) | 0.463 |
| Model 2 | 0.98 (0.87; 1.11) | 1.00 (0.85; 1.18) | 0.930 | 1.07 (0.91; 1.25) | 0.96 (0.75; 1.23) | 0.903 | 0.88 (0.72; 1.07) | 1.00 (0.80; 1.25) | 0.794 |
| **Former smoker** | *5,088/29,063* |  |  |  |  |  |  |  |  |
| Unadjusted | 0.96 (0.90; 1.02) | **0.88 (0.82; 0.95)** | **<0.001** | 1.01 (0.94; 1.09) | 0.95 (0.86; 1.04) | 0.296 | 0.93 (0.83; 1.05) | 0.94 (0.84; 1.06) | 0.319 |
| Model 1 | 0.97 (0.91; 1.04) | **0.93 (0.86; 0.99)** | **0.037** | 1.00 (0.92; 1.08) | 0.92 (0.84; 1.01) | 0.093 | 0.93 (0.82; 1.04) | 0.93 (0.83; 1.04) | 0.225 |
| Model 2 | 1.00 (0.94; 1.07) | 0.98 (0.91; 1.05) | 0.600 | 1.02 (0.95; 1.11) | 0.97 (0.88; 1.06) | 0.573 | 0.94 (0.84; 1.06) | 0.98 (0.88; 1.11) | 0.832 |

Model 1 adjusted for age. Model 2 additionally adjusted for trial arm, race, education, smoking status, cigarette, cigar or pipe smoking. Bold represents p<0.05 for tertile vs. tertile 1.

**Supplementary Table 4 Associations between 2018 WCRF/AICR Score and risk of 17 cancer sites reviewed by the WCRF/AICR, stratified by sex and smoking status (per 1-point increment in score), in the Prostate, Lung, Colorectal, and Ovarian Cancer Screening Trial**

|  | **All** | | **Males** | | **Females** | |
| --- | --- | --- | --- | --- | --- | --- |
|  | **HR (95% CI)** | **P-value** | **HR (95% CI)** | **P-value** | **HR (95% CI)** | **P-value** |
| **All**  8,821/68,223 |  |  | 4,938/31,550 |  | 3,881/36,663 |  |
| Unadjusted | **0.91 (0.89; 0.93)** | **<0.001** | **0.96 (0.93; 0.99)** | **0.006** | **0.92 (0.89; 0.95)** | **<0.001** |
| Model 1 | **0.93 (0.92; 0.95)** | **<0.001** | **0.95 (0.93; 0.98)** | **0.001** | **0.91 (0.89; 0.94)** | **<0.001** |
| Model 2 | **0.97 (0.95; 0.99)** | **0.007** | 1.00 (0.97; 1.02) | 0.762 | **0.91 (0.89; 0.94)** | **<0.001** |
| **Never smoker**  3,681/33,715 |  |  | 1,708/12,367 |  | 1,973/21,348 |  |
| Unadjusted | **0.93 (0.91; 0.96)** | **<0.001** | 1.00 (0.95; 1.05) | 0.975 | **0.93 (0.89; 0.97)** | **<0.001** |
| Model 1 | **0.95 (0.92; 0.98)** | **0.003** | 0.99 (0.95; 1.04) | 0.743 | **0.92 (0.89; 0.96)** | **<0.001** |
| Model 2 | **0.95 (0.92; 0.98)** | **0.001** | 0.99 (0.94; 1.14) | 0.648 | **0.92 (0.88; 0.96)** | **<0.001** |
| **Current smoker**  1,100/5,445 |  |  | 640/2,705 |  | 460/2,738 |  |
| Unadjusted | 0.95 (0.90; 1.01) | 0.129 | 1.00 (0.93; 1.10) | 0.864 | 0.97 (0.88; 1.06) | 0.488 |
| Model 1 | 0.97 (0.92; 1.04) | 0.420 | 0.99 (0.91; 1.08) | 0.784 | 0.96 (0.87; 1.05) | 0.370 |
| Model 2 | 1.00 (0.94; 1.07) | 0.879 | 1.02 (0.94; 1.11) | 0.626 | 0.99 (0.90; 1.08) | 0.759 |
| **Former smoker**  4,039/29,058 |  |  | 2,590/16,471 |  | 1,450/12,587 |  |
| Unadjusted | **0.94 (0.91; 0.97)** | **<0.001** | 0.98 (0.94; 1.02) | 0.257 | **0.95 (0.90; 0.99)** | **0.037** |
| Model 1 | **0.96 (0.93; 0.99)** | **0.007** | 0.97 (0.93; 1.01) | 0.116 | **0.94 (0.90; 0.99)** | **0.019** |
| Model 2 | 0.99 (0.96; 1.02) | 0.397 | 0.99 (0.96; 1.04) | 0.785 | 0.97 (0.93; 1.02) | 0.299 |

Model 1 adjusted for age, sex (if applicable). Model 2 adjusted for age, sex (if applicable), trial arm, race, education, smoking status, cigarette, cigar or pipe smoking.

**Supplementary Table 5 Risk of 17 cancer sites reviewed by the WCRF/AICR according to 2018 WCRF/AICR Score tertiles, stratified by smoking status in the Prostate, Lung, Colorectal, and Ovarian Cancer Screening Trial**

|  | **Tertile 1** | **Tertile 2**  **(3.25 – 4 points)** | **Tertile 3**  **(4.25 – 7 points)** |  |
| --- | --- | --- | --- | --- |
| **All**  8,821/68,223 | **HR (95% CI)** | **HR (95% CI)** | **HR (95% CI)** | **P trend** |
| Unadjusted | Ref | 0.95 (0.91; 1.01) | **0.81 (0.76; 0.85)** | **<0.001** |
| Model 1 | Ref | 0.98 (0.93; 1.03) | **0.86 (0.81; 0.91)** | **<0.001** |
| Model 2 | Ref | 1.03 (0.98; 1.08) | **0.94 (0.89; 0.99)** | **0.048** |
| **Never smoker**  3,681/33,715 |  |  |  |  |
| Unadjusted | Ref | 1.04 (0.96; 1.12) | **0.86 (0.80; 0.94)** | **<0.001** |
| Model 1 | Ref | 1.04 (0.97; 1.13) | **0.91 (0.84; 0.99)** | **0.018** |
| Model 2 | Ref | 1.04 (0.96; 1.13) | **0.90 (0.83; 0.98)** | **0.009** |
| **Current smoker**  1,100/5,445 |  |  |  |  |
| Unadjusted | Ref | 0.94 (0.82; 1.07) | 0.90 (0.76; 1.06) | 0.169 |
| Model 1 | Ref | 0.95 (0.83; 1.08) | 0.95 (0.80; 1.13) | 0.446 |
| Model 2 | Ref | 0.98 (0.86; 1.12) | 1.03 (0.86; 1.22) | 0.889 |
| **Former smoker** |  |  |  |  |
| Unadjusted | Ref | 0.97 (0.91; 1.05) | **0.87 (0.80; 0.94)** | **0.001** |
| Model 1 | Ref | 0.99 (0.92; 1.06) | **0.92 (0.85; 0.99)** | **0.035** |
| Model 2 | Ref | 1.02 (0.95; 1.10) | 0.98 (0.90; 1.06) | 0.660 |

Model 1 adjusted for age and sex. Model 2 adjusted for age, sex, trial arm, race, education, smoking status, cigarette, cigar or pipe smoking. Bold represents p<0.05 for tertile vs. tertile 1.

**Supplementary Table 6 Associations between 2018 WCRF/AICR Score and risk of individual cancer sites in the Prostate, Lung, Colorectal, and Ovarian Cancer Screening Trial**

|  | **All** | | **Males** | | **Females** | |
| --- | --- | --- | --- | --- | --- | --- |
|  | **HR (95% CI)** | **P-value** | **HR (95% CI)** | **P-value** | **HR (95% CI)** | **P-value** |
| **Hematopoietic** | 1,384/68,223 |  | 805/31,550 |  | 579/36,673 |  |
| Unadjusted | 0.95 (0.91; 1.00) | 0.055 | 0.98 (0.91; 1.05) | 0.486 | 1.01 (0.94; 1.09) | 0.738 |
| Model 1 | 0.97 (0.92; 1.02) | 0.255 | 0.95 (0.89; 1.02) | 0.146 | 1.00 (0.92; 1.08) | 0.932 |
| Model 2 | 0.98 (0.93; 1.04) | 0.512 | 0.97 (0.90; 1.04) | 0.379 | 1.00 (0.92; 1.08) | 0.981 |
| **Breast** |  |  |  |  | 1,646/36,673 |  |
| Unadjusted |  |  |  |  | **0.91 (0.87; 0.95)** | **<0.001** |
| Model 1 |  |  |  |  | **0.91 (0.87; 0.96)** | **<0.001** |
| Model 2 |  |  |  |  | **0.91 (0.87; 0.96)** | **<0.001** |
| Model 3 |  |  |  |  | **0.91 (0.87; 0.96)** | **<0.001** |
| **Breast**  ***Score inc. breastfeeding*** |  |  |  |  | 1,638/36,418 |  |
| Unadjusted |  |  |  |  | **0.93 (0.89; 0.97)** | **0.001** |
| Model 1 |  |  |  |  | **0.94 (0.90; 0.97)** | **0.001** |
| Model 2 |  |  |  |  | **0.94 (0.90; 0.98)** | **0.002** |
| Model 3 |  |  |  |  | **0.93 (0.89; 0.98)** | **0.002** |
| **Prostate** |  |  | 2,053/31,550 |  |  |  |
| Unadjusted |  |  | 1.00 (0.96; 1.05) | 0.838 |  |  |
| Model 1 |  |  | 1.02 (0.98; 1.06) | 0.408 |  |  |
| Model 2 |  |  | 1.00 (0.96; 1.05) | 0.834 |  |  |
| **Lung** | 1,214/68,223 |  | 665/31,550 |  | 549/36,673 |  |
| Unadjusted | **0.81 (0.77; 0.85)** | **<0.001** | **0.82 (0.76; 0.89)** | **<0.001** | **0.84 (0.78; 0.91)** | **<0.001** |
| Model 1 | **0.81 (0.77; 0.86)** | **<0.001** | **0.80 (0.74; 0.87)** | **<0.001** | **0.83 (0.76; 0.89)** | **<0.001** |
| Model 2 | 1.03 (0.98; 1.10) | 0.247 | 1.03 (0.95; 1.12) | 0.422 | 1.04 (0.95; 1.13) | 0.398 |
| *Never smokers* |  |  |  |  |  |  |
| Unadjusted | 1.05 (0.89; 1.24) | 0.588 | 1.03 (0.77; 1.37) | 0.858 | 1.06 (0.86; 1.31) | 0.564 |
| Model 1 | 1.01 (0.86; 1.20) | 0.866 | 0.97 (0.73; 1.30) | 0.843 | 1.04 (0.84; 1.28) | 0.730 |
| Model 2 | 1.01 (0.85; 1.20) | 0.911 | 1.02 (0.76; 1.38) | 0.882 | 1.01 (0.82; 1.25) | 0.916 |
| *Current smokers* |  |  |  |  |  |  |
| Unadjusted | 0.99 (0.90; 1.09) | 0.808 | 1.01 (0.88; 1.16) | 0.851 | 1.01 (0.88; 1.16) | 0.866 |
| Model 1 | 0.99 (0.90; 1.09) | 0.845 | 0.99 (0.87; 1.14) | 0.909 | 0.99 (0.87; 1.14) | 0.908 |
| Model 2 | 1.06 (0.96; 1.17) | 0.222 | 1.07 (0.93; 1.23) | 0.354 | 1.06 (0.93; 1.22) | 0.381 |
| *Former smokers* |  |  |  |  |  |  |
| Unadjusted | **0.92 (0.85; 0.99)** | **0.036** | 0.94 (0.85; 1.04) | 0.259 | 0.92 (0.82; 1.03) | 0.144 |
| Model 1 | **0.90 (0.84; 0.98)** | **0.011** | 0.91 (0.82; 1.01) | 0.082 | 0.90 (0.80; 1.01) | 0.064 |
| Model 2 | 1.02 (0.94; 1.10) | 0.622 | 1.02 (0.92; 1.13) | 0.751 | 1.02 (0.91; 1.15) | 0.730 |
| **Melanoma** | 951/68,223 |  | 610/31,550 |  | 341/36,663 |  |
| Unadjusted | 1.03 (0.97; 1.10) | 0.318 | **1.13 (1.05; 1.22)** | **0.002** | 1.05 (0.95; 1.16) | 0.307 |
| Model 1 | **1.09 (1.02; 1.16)** | **0.006** | **1.11 (1.03; 1.20)** | **0.008** | 1.05 (0.95; 1.16) | 0.314 |
| Model 2 | 1.06 (0.99; 1.12) | 0.094 | 1.07 (0.99; 1.16) | 0.092 | 1.03 (0.93; 1.14) | 0.600 |
| **Bladder** | 679/68,223 |  | 529/31,550 |  | 150/36,673 |  |
| Unadjusted | **0.82 (0.77; 0.89)** | **<0.001** | 0.95 (0.87; 1.04) | 0.258 | **0.79 (0.68; 0.92)** | **0.003** |
| Model 1 | **0.88 (0.82; 0.95)** | **0.001** | **0.91 (0.84; 0.99)** | **0.040** | **0.78 (0.67; 0.91)** | **0.001** |
| Model 2 | 0.95 (0.87; 1.02) | 0.152 | 0.99 (0.90; 1.08) | 0.787 | **0.82 (0.70; 0.96)** | **0.016** |
| **Colorectal** | 586/68,223 |  | 291/31,550 |  | 295/36,673 |  |
| Unadjusted | 0.95 (0.88; 1.03) | 0.203 | 0.98 (0.87; 1.09) | 0.675 | 0.95 (0.86; 1.06) | 0.389 |
| Model 1 | 0.94 (0.87; 1.01) | 0.107 | 0.94 (0.84; 1.05) | 0.282 | 0.93 (0.84; 1.04) | 0.222 |
| Model 2 | 0.98 (0.90; 1.06) | 0.617 | 0.98 (0.87; 1.11) | 0.759 | 0.97 (0.87; 1.09) | 0.635 |
| **Pancreatic** | 341/68,223 |  | 155/31,550 |  | 186/36,673 |  |
| Unadjusted | **0.88 (0.79; 0.97)** | **0.011** | **0.83 (0.71; 0.98)** | **0.026** | 0.91 (0.79; 1.04) | 0.150 |
| Model 1 | **0.86 (0.77; 0.95)** | **0.004** | **0.82 (0.69; 0.96)** | **0.013** | 0.89 (0.78; 1.02) | 0.092 |
| Model 2 | **0.86 (0.78; 0.96)** | **0.007** | **0.82 (0.69; 0.96)** | **0.017** | 0.90 (0.78; 1.04) | 0.144 |
| **Renal** | 308/68,223 |  | 190/31,550 |  | 118/36,673 |  |
| Unadjusted | **0.85 (0.76; 0.94)** | **0.003** | **0.85 (0.73; 0.98)** | **0.024** | 0.95 (0.80; 1.13) | 0.563 |
| Model 1 | **0.88 (0.79; 0.99)** | **0.027** | **0.84 (0.72; 0.97)** | **0.017** | 0.95 (0.80; 1.13) | 0.549 |
| Model 2 | 0.93 (0.83; 1.04) | 0.203 | 0.90 (0.77; 1.03) | 0.124 | 0.99 (0.83; 1.18) | 0.892 |
| **Head & Neck** | 240/68,223 |  | 177/31,550 |  | 63/36,673 |  |
| Unadjusted | **0.81 (0.72; 0.92)** | **0.001** | **0.84 (0.72; 0.98)** | **0.023** | 0.99 (0.79; 1.25) | 0.930 |
| Model 1 | **0.88 (0.78; 0.99)** | **0.048** | **0.84 (0.72; 0.97)** | **0.021** | 0.99 (0.79; 1.25) | 0.952 |
| Model 2 | 0.99 (0.87; 1.12) | 0.827 | 0.94 (0.81; 1.10) | 0.453 | 1.09 (0.85; 1.38) | 0.505 |
| **Upper GI** | 243/68,223 |  | 184/31,550 |  | 59/36,673 |  |
| Unadjusted | **0.80 (0.71; 0.91)** | **<0.001** | 0.89 (0.77; 1.03) | 0.107 | 0.85 (0.67; 1.09) | 0.199 |
| Model 1 | **0.85 (0.75; 0.97)** | **0.014** | 0.87 (0.75; 1.01) | 0.059 | 0.82 (0.64; 1.05) | 0.120 |
| Model 2 | 0.91 (0.80; 1.03) | 0.141 | 0.93 (0.80; 1.08) | 0.330 | 0.85 (0.66; 1.09) | 0.191 |
| **Glioma** | 131/68,223 |  | 68/31,550 |  | 63/36,673 |  |
| Unadjusted | 0.93 (0.79; 1.09) | 0.377 | 0.92 (0.73; 1.17) | 0.497 | 0.98 (0.77; 1.23) | 0.842 |
| Model 1 | 0.93 (0.79; 1.10) | 0.428 | 0.91 (0.72; 1.16) | 0.449 | 0.96 (0.76; 1.21) | 0.733 |
| Model 2 | 0.92 (0.78; 1.10) | 0.366 | 0.91 (0.71; 1.17) | 0.471 | 0.94 (0.74; 1.19) | 0.585 |
| **Thyroid** | 120/68,223 |  | 36/31,550 |  | 84/36,673 |  |
| Unadjusted | 1.03 (0.87; 1.22) | 0.748 | 0.78 (0.56; 1.09) | 0.144 | 1.07 (0.87; 1.30) | 0.523 |
| Model 1 | 1.00 (0.84; 1.18) | 0.982 | 0.80 (0.57; 1.12) | 0.201 | 1.08 (0.89; 1.32) | 0.443 |
| Model 2 | 0.98 (0.82; 1.17) | 0.834 | 0.77 (0.54; 1.08) | 0.129 | 1.07 (0.88; 1.32) | 0.496 |
| **Liver** | 96/68,223 |  | 60/31,550 |  | 36/36,673 |  |
| Unadjusted | 0.92 (0.76; 1.12) | 0.415 | 0.92 (0.72; 1.19) | 0.540 | 1.06 (0.78; 1.43) | 0.730 |
| Model 1 | 0.95 (0.78; 1.15) | 0.584 | 0.89 (0.69; 1.15) | 0.379 | 1.03 (0.76; 1.41) | 0.837 |
| Model 2 | 1.00 (0.82; 1.22) | 0.989 | 0.97 (0.75; 1.27) | 0.844 | 1.03 (0.75; 1.41) | 0.846 |
| **Ovarian** |  |  |  |  | 187/36,673 |  |
| Unadjusted |  |  |  |  | 1.02 (0.89; 1.16) | 0.826 |
| Model 1 |  |  |  |  | 1.01 (0.88; 1.16) | 0.872 |
| Model 2 |  |  |  |  | 1.01 (0.88; 1.16) | 0.907 |
| Model 3 |  |  |  |  | 1.00 (0.88; 1.15) | 0.990 |
| **Endometrial^a^** |  |  |  |  | 260/23,448 |  |
| Unadjusted |  |  |  |  | 0.93 (0.83; 1.04) | 0.209 |
| Model 1 |  |  |  |  | 0.93 (0.83; 1.04) | 0.220 |
| Model 2 |  |  |  |  | 0.90 (0.80; 1.01) | 0.085 |
| Model 3 |  |  |  |  | 0.90 (0.80; 1.01) | 0.078 |

Model 1 adjusted for: age and sex (if applicable). Model 2 adjusted for: age, sex, trial arm, race, education, smoking status, cigarette, cigar/pipe smoking status. Model 3 (female cancers): additionally adjusted for female hormone use, parity, contraceptive use, family history of that cancer. ^a^participants with prior hysterectomy were excluded.

**Supplementary Table 7 Risk of individual cancer sites according to 2018 WCRF/AICR Score tertiles in the Prostate, Lung, Colorectal, and Ovarian Cancer Screening Trial**

|  | **All** | | | **Males** | | | **Females** | | |
| --- | --- | --- | --- | --- | --- | --- | --- | --- | --- |
|  | **Tertile 2**  **(3.25 – 4 points)** | **Tertile 3**  **(4.25 – 7 points)** |  | **Tertile 2**  **(3.25 – 4 points)** | **Tertile 3**  **(4.25 – 7 points)** |  | **Tertile 2**  **(3.25 – 4 points)** | **Tertile 3**  **(4.25 – 7 points)** |  |
|  | **HR (95% CI)** | **HR (95% CI)** | **P trend** | **HR (95% CI)** | **HR (95% CI)** | **P trend** | **HR (95% CI)** | **HR (95% CI)** | **P trend** |
| **Prostate**  2,053/31,550 |  |  |  |  |  |  |  |  |  |
| Unadjusted |  |  |  | 1.03 (0.93; 1.14) | 1.02 (0.91; 1.14) | 0.729 |  |  |  |
| Model 1 |  |  |  | 1.05 (0.95; 1.16) | 1.05 (0.94; 1.17) | 0.355 |  |  |  |
| Model 2 |  |  |  | 1.03 (0.93; 1.14) | 1.02 (0.91; 1.14) | 0.712 |  |  |  |
| **Breast**  1,646/36,673 |  |  |  |  |  |  |  |  |  |
| Unadjusted |  |  |  |  |  |  | 0.92 (0.82; 1.05) | **0.81 (0.71; 0.91)** | **<0.001** |
| Model 1 |  |  |  |  |  |  | 0.93 (0.83; 1.05) | **0.81 (0.72; 0.92)** | **0.001** |
| Model 2 |  |  |  |  |  |  | 0.93 (0.83; 1.05) | **0.81 (0.72; 0.92)** | **0.001** |
| Model 3 |  |  |  |  |  |  | 0.94 (0.83; 1.06) | **0.81 (0.72; 0.92)** | **0.001** |
| **Breast**  ***(breastfeeding)*** |  |  |  |  |  |  |  |  |  |
| Unadjusted |  |  |  |  |  |  | 0.95 (0.84; 1.06) | **0.84 (0.74; 0.94)** | **0.004** |
| Model 1 |  |  |  |  |  |  | 0.95 (0.85; 1.06) | **0.84 (0.75; 0.95)** | **0.007** |
| Model 2 |  |  |  |  |  |  | 0.95 (0.85; 1.07) | **0.85 (0.75; 0.96)** | **0.010** |
| Model 3 |  |  |  |  |  |  | 0.95 (0.85; 1.07) | **0.84 (0.74; 0.96)** | **0.009** |
| **Hematopoietic**  1,384/68,223 |  |  |  |  |  |  |  |  |  |
| Unadjusted | **0.88 (0.77; 0.99)** | **0.87 (0.76; 0.99)** | **0.027** | 0.91 (0.77; 1.06) | 0.92 (0.77; 1.10) | 0.298 | 0.91 (0.74; 1.12) | 1.00 (0.82; 1.22) | 0.951 |
| Model 1 | 0.88 (0.77; 1.00) | 0.91 (0.80; 1.04) | 0.135 | 0.88 (0.75; 1.03) | 0.86 (0.72; 1.03) | 0.082 | 0.89 (0.72; 1.09) | 0.96 (0.79; 1.17) | 0.778 |
| Model 2 | 0.89 (0.78; 1.01) | 0.93 (0.82; 1.07) | 0.278 | 0.90 (0.76; 1.05) | 0.90 (0.75; 1.08) | 0.211 | 0.89 (0.72; 1.09) | 0.97 (0.79; 1.18) | 0.807 |
| **Lung**  1,214/68,223 |  |  |  | 665/31,550 |  |  | 549/36,673 |  |  |
| Unadjusted | 0.88 (0.77; 1.00) | **0.59 (0.51; 0.69)** | **<0.001** | 0.94 (0.80; 1.11) | **0.58 (0.46; 0.72)** | **<0.001** | 0.85 (0.69; 1.04) | **0.67 (0.55; 0.83)** | **<0.001** |
| Model 1 | **0.88 (0.77; 0.99)** | **0.61 (0.52; 0.70)** | **<0.001** | 0.92 (0.78; 1.09) | **0.55 (0.44; 0.69)** | **<0.001** | 0.83 (0.68; 1.01) | **0.65 (0.53; 0.80)** | **<0.001** |
| Model 2 | 1.14 (1.00; 1.30) | 1.08 (0.93; 1.26) | 0.188 | **1.25 (1.05; 1.47)** | 1.00 (0.79; 1.25) | 0.447 | 1.03 (0.85; 1.27) | 1.13 (0.91; 1.40) | 0.277 |
| *Never smokers* |  |  |  |  |  |  |  |  |  |
| Unadjusted | 1.42 (0.90; 2.23) | 1.18 (0.74; 1.87) | 0.571 | 1.28 (0.64; 2.55) | 1.06 (0.49; 2.55) | 0.848 | 1.54 (0.84; 2.82) | 1.28 (0.70; 2.33) | 0.548 |
| Model 1 | 1.35 (0.86; 2.12) | 1.09 (0.68; 1.74) | 0.816 | 1.19 (0.60; 2.38) | 0.92 (0.43; 2.01) | 0.862 | 1.48 (0.81; 2.71) | 1.21 (0.66; 2.21) | 0.683 |
| Model 2 | 1.35 (0.86; 2.13) | 1.08 (0.67; 1.74) | 0.846 | 1.27 (0.63; 2.54) | 1.05 (0.48; 2.30) | 0.880 | 1.46 (0.80; 2.67) | 1.14 (0.62; 2.09) | 0.849 |
| *Current smokers* |  |  |  |  |  |  |  |  |  |
| Unadjusted | 0.93 (0.75; 1.14) | 0.96 (0.74; 1.24) | 0.609 | 1.00 (0.76; 1.32) | 0.87 (0.57; 1.33) | 0.628 | 0.89 (0.65; 1.22) | 1.10 (0.79; 1.55) | 0.707 |
| Model 1 | 0.91 (0.74; 1.12) | 0.97 (0.74; 1.25) | 0.611 | 0.98 (0.74; 1.28) | 0.82 (0.54; 1.26) | 0.445 | 0.84 (0.61; 1.15) | 1.05 (0.75; 1.48) | 0.946 |
| Model 2 | 0.98 (0.80; 1.21) | 1.15 (0.88; 1.50) | 0.427 | 1.08 (0.82; 1.43) | 0.99 (0.64; 1.52) | 0.811 | 0.89 (0.65; 1.23) | 1.24 (0.88; 1.76) | 0.341 |
| *Former smokers* |  |  |  |  |  |  |  |  |  |
| Unadjusted | 1.13 (0.95; 1.36) | 0.84 (0.69; 1.03) | 0.150 | **1.26 (1.01; 1.58)** | 0.85 (0.64; 1.14) | 0.574 | 0.97 (0.72; 1.30) | 0.84 (0.62; 1.14) | 0.250 |
| Model 1 | 1.11 (0.92; 1.32) | 0.81 (0.66; 1.00) | 0.077 | 1.22 (0.97; 1.53) | 0.79 (0.60; 1.06) | 0.272 | 0.94 (0.70; 1.27) | 0.80 (0.59; 1.09) | 0.158 |
| Model 2 | **1.25 (1.04; 1.50)** | 1.08 (0.88; 1.34) | 0.351 | **1.38 (1.10; 1.73)** | 1.03 (0.76; 1.37) | 0.436 | 1.06 (0.79; 1.43) | 1.09 (0.80; 1.49) | 0.589 |
| **Melanoma**  951/68,223 |  |  |  |  |  |  |  |  |  |
| Unadjusted | **1.19 (1.02; 1.38)** | 1.07 (0.91; 1.26) | 0.379 | **1.27 (1.05; 1.53)** | **1.28 (1.05; 1.58)** | **0.011** | 1.20 (0.91; 1.59) | 1.19 (0.91; 1.56) | 0.247 |
| Model 1 | **1.23 (1.06; 1.44)** | 1.22 (1.04; 1.44) | **0.015** | **1.24 (1.03; 1.49)** | **1.23 (1.00; 1.51)** | **0.034** | 1.20 (0.91; 1.59) | 1.19 (0.90; 1.56) | 0.248 |
| Model 2 | **1.18 (1.01; 1.37)** | 1.12 (0.95; 1.33) | 0.166 | 1.18 (0.98; 1.42) | 1.12 (0.91; 1.38) | 0.239 | 1.16 (0.88; 1.53) | 1.11 (0.84; 1.46) | 0.523 |
| **Bladder**  679/68,223 |  |  |  |  |  |  |  |  |  |
| Unadjusted | **0.78 (0.66; 0.93)** | **0.64 (0.52; 0.77)** | **<0.001** | 0.93 (0.76; 1.13) | 0.93 (0.74; 1.16) | 0.461 | **0.59 (0.40; 0.87)** | **0.53 (0.36; 0.79)** | **0.001** |
| Model 1 | **0.81 (0.68; 0.97)** | **0.75 (0.62; 0.91)** | **0.003** | 0.89 (0.73; 1.08) | 0.84 (0.68; 1.05) | 0.113 | **0.58 (0.39; 0.85)** | **0.51 (0.35; 0.76)** | **0.001** |
| Model 2 | 0.88 (0.74; 1.05) | 0.89 (0.73; 1.09) | 0.209 | 0.97 (0.79; 1.18) | 1.01 (0.81; 1.27) | 0.972 | **0.61 (0.42; 0.89)** | **0.59 (0.40; 0.88)** | **0.008** |
| **Colorectal**  586/68,223 |  |  |  | 291/31,550 |  |  | 295/36,673 |  |  |
| Unadjusted | 1.16 (0.95; 1.40) | 0.99 (0.81; 1.22) | 0.963 | 1.13 (0.87; 1.47) | 1.11 (0.82; 1.49) | 0.459 | 1.21 (0.91; 1.61) | 0.97 (0.73; 1.31) | 0.760 |
| Model 1 | 1.13 (0.93; 1.37) | 0.96 (0.78; 1.19) | 0.756 | 1.08 (0.83; 1.41) | 1.01 (0.75; 1.36) | 0.889 | 1.18 (0.88; 1.57) | 0.93 (0.70; 1.25) | 0.553 |
| Model 2 | 1.19 (0.98; 1.45) | 1.08 (0.87; 1.34) | 0.446 | 1.15 (0.83; 1.54) | 1.13 (0.83; 1.54) | 0.374 | 1.23 (0.92; 1.65) | 1.04 (0.77; 1.40) | 0.896 |
| **Pancreatic**  341/68,223 |  |  |  |  |  |  |  |  |  |
| Unadjusted | 1.01 (0.79; 1.30) | **0.72 (0.54; 0.95)** | **0.023** | 0.87 (0.62; 1.24) | **0.61 (0.39; 0.96)** | **0.034** | 1.18 (0.83; 1.68) | 0.82 (0.56; 1.19) | 0.252 |
| Model 1 | 0.99 (0.77; 1.26) | **0.69 (0.52; 0.91)** | **0.010** | 0.85 (0.60; 1.21) | **0.58 (0.37; 0.91)** | **0.019** | 1.16 (0.81; 1.64) | 0.79 (0.54; 1.15) | 0.180 |
| Model 2 | 1.00 (0.78; 1.28) | **0.70 (0.53; 0.93)** | **0.018** | 0.85 (0.60; 1.21) | **0.59 (0.37; 0.93)** | **0.024** | 1.17 (0.82; 1.67) | 0.82 (0.56; 1.20) | 0.266 |
| **Renal**  308/68,223 |  |  |  |  |  |  |  |  |  |
| Unadjusted | **0.70 (0.54; 0.92)** | **0.69 (0.52; 0.90)** | **0.005** | 0.73 (0.53; 1.02) | 0.74 (0.51; 1.08) | 0.453 | 0.73 (0.47; 1.16) | 0.81 (0.53; 1.25) | 0.374 |
| Model 1 | **0.73 (0.56; 0.95)** | 0.76 (0.58; 1.01) | **0.039** | 0.72 (0.52; 1.00) | 0.72 (0.50; 1.05) | 0.564 | 0.73 (0.47; 1.15) | 0.81 (0.53; 1.25) | 0.365 |
| Model 2 | 0.77 (0.59; 1.01) | 0.86 (0.65; 1.15) | 0.233 | 0.78 (0.52; 1.00) | 0.84 (0.57; 1.23) | 0.450 | 0.77 (0.49; 1.22) | 0.89 (0.57; 1.39) | 0.634 |
| **Head & Neck**  240/68,223 |  |  |  |  |  |  |  |  |  |
| Unadjusted | 0.78 (0.58; 1.04) | **0.54 (0.39; 0.75)** | **<0.001** | 0.82 (0.59; 1.13) | **0.58 (0.38; 0.88)** | **0.009** | 0.93 (0.50; 1.72) | 0.91 (0.50; 1.67) | 0.766 |
| Model 1 | 0.83 (0.62; 1.11) | **0.67 (0.48; 0.93)** | **0.017** | 0.81 (0.59; 1.13) | **0.57 (0.38; 0.87)** | **0.008** | 0.93 (0.50; 1.73) | 0.92 (0.50; 1.68) | 0.783 |
| Model 2 | 0.95 (0.71; 1.27) | 0.87 (0.61; 1.22) | 0.424 | 0.93 (0.67; 1.29) | 0.75 (0.49; 1.15) | 0.202 | 1.02 (0.55; 1.91) | 1.15 (0.61; 2.15) | 0.664 |
| **Upper GI**  243/68,223 |  |  |  |  |  |  |  |  |  |
| Unadjusted | 0.96 (0.72; 1.27) | **0.53 (0.38; 0.75)** | **0.001** | 0.90 (0.65; 1.24) | 0.72 (0.49; 1.06) | 0.103 | 1.58 (0.86; 2.89) | 0.62 (0.30; 1.28) | 0.181 |
| Model 1 | 0.99 (0.75; 1.32) | **0.63 (0.44; 0.89)** | **0.015** | 0.88 (0.63; 1.21) | 0.68 (0.46; 1.01) | 0.060 | 1.50 (0.82; 2.76) | 0.57 (0.28; 1.20) | 0.120 |
| Model 2 | 1.07 (0.80; 1.42) | 0.72 (0.51; 1.03) | 0.120 | 0.95 (0.68; 1.32) | 0.80 (0.53; 1.19) | 0.294 | 1.55 (0.84; 2.86) | 0.61 (0.29; 1.30) | 0.190 |
| **Glioma**  131/68,223 |  |  |  |  |  |  |  |  |  |
| Unadjusted | 1.06 (0.71; 1.59) | 0.84 (0.54; 1.30) | 0.454 | 0.90 (0.52; 1.56) | 0.99 (0.54; 1.80) | 0.916 | 1.33 (0.73; 2.44) | 0.84 (0.44; 1.62) | 0.547 |
| Model 1 | 1.06 (0.71; 1.59) | 0.86 (0.55; 1.33) | 0.511 | 0.89 (0.51; 1.54) | 0.97 (0.53; 1.77) | 0.862 | 1.30 (0.71; 2.38) | 0.81 (0.42; 1.56) | 0.472 |
| Model 2 | 1.04 (0.69; 1.55) | 0.83 (0.53; 1.31) | 0.445 | 0.89 (0.51; 1.55) | 0.98 (0.53; 1.83) | 0.908 | 1.23 (0.67; 2.27) | 0.76 (0.39; 1.47) | 0.356 |
| **Thyroid**  120/68,223 |  |  |  |  |  |  |  |  |  |
| Unadjusted | 1.08 (0.70; 1.66) | 1.01 (0.64; 1.58) | 0.966 | 1.32 (0.65; 2.68) | 0.58 (0.21; 1.61) | 0.453 | 0.91 (0.53; 1.57) | 0.98 (0.58; 1.65) | 0.953 |
| Model 1 | 1.06 (0.69; 1.64) | 0.94 (0.59; 1.48) | 0.778 | 1.37 (0.68; 2.79) | 0.63 (0.22; 1.74) | 0.564 | 0.93 (0.54; 1.60) | 1.01 (0.60; 1.70) | 0.964 |
| Model 2 | 1.04 (0.68; 1.61) | 0.90 (0.56; 1.43) | 0.647 | 1.33 (0.65; 2.72) | 0.57 (0.20; 1.62) | 0.450 | 0.91 (0.53; 1.58) | 0.98 (0.57; 1.67) | 0.960 |
| **Liver**  96/68,223 |  |  |  |  |  |  |  |  |  |
| Unadjusted | 1.04 (0.65; 1.69) | 0.95 (0.58; 1.57) | 0.851 | 1.04 (0.59; 1.85) | 0.88 (0.45; 1.72) | 0.747 | 1.25 (0.51; 3.07) | 1.50 (0.64; 3.49) | 0.348 |
| Model 1 | 1.05 (0.65; 1.70) | 1.02 (0.61; 1.69) | 0.940 | 1.00 (0.56; 1.77) | 0.81 (0.41; 1.59) | 0.572 | 1.21 (0.50; 2.97) | 1.43 (0.61; 3.34) | 0.408 |
| Model 2 | 1.12 (0.69; 1.81) | 1.17 (0.69; 1.97) | 0.561 | 1.11 (0.62; 1.99) | 1.00 (0.50; 2.01) | 0.938 | 1.21 (0.49; 2.98) | 1.43 (0.60; 3.40) | 0.411 |
| **Endometrial**^a^  260/23,448 |  |  |  |  |  |  |  |  |  |
| Unadjusted |  |  |  |  |  |  | 0.74 (0.55; 1.01) | 0.83 (0.62; 1.11) | 0.245 |
| Model 1 |  |  |  |  |  |  | 0.75 (0.55; 1.01) | 0.83 (0.62; 1.11) | 0.255 |
| Model 2 |  |  |  |  |  |  | **0.73 (0.53; 0.99)** | 0.77 (0.57; 1.04) | 0.108 |
| Model 3 |  |  |  |  |  |  | 0.73 (0.54; 1.00) | 0.77 (0.57; 1.04) | 0.102 |
| **Ovarian**  187/36,673 |  |  |  |  |  |  |  |  |  |
| Unadjusted |  |  |  |  |  |  | 1.31 (0.91; 1.88) | 1.08 (0.74; 1.57) | 0.786 |
| Model 1 |  |  |  |  |  |  | 1.30 (0.90; 1.87) | 1.07 (0.74; 1.56) | 0.823 |
| Model 2 |  |  |  |  |  |  | 1.29 (0.89; 1.87) | 1.06 (0.73; 1.55) | 0.864 |
| Model 3 |  |  |  |  |  |  | 1.27 (0.88; 1.84) | 1.04 (0.71; 1.52) | 0.964 |

Model 1 adjusted for age and sex (if applicable). Model 2 adjusted for age, sex (if applicable), trial arm, race, education, smoking status, cigarette, cigar or pipe smoking.

Bold represents p<0.05 for tertile vs. tertile 1. Model 3 (female cancers): additionally adjusted for female hormone use, parity, contraceptive use, family history of that cancer. ^a^participants with prior hysterectomy were excluded.

**Supplementary Table 8 2018 WCRF/Score and risk of** **prostate, lung, colorectal, and ovarian cancers, stratified according to trial arm, in the Prostate, Lung, Colorectal, and Ovarian Cancer Screening Trial**

|  | **Continuous** | | **Tertile 2**  **(3.25 – 4 points)** | **Tertile 3**  **(4.25 – 7 points)** |  |  |
| --- | --- | --- | --- | --- | --- | --- |
|  | **HR (95% CI)** | **P-value** | **HR (95% CI)** | **HR (95% CI)** | **P trend** | **P heterogeneity** |
| **Overall** |  |  |  |  |  | 0.513 |
| *Intervention* |  |  |  |  |  |  |
| Unadjusted | **0.92 (0.89; 0.94)** | **<0.001** | 0.95 (0.90; 1.01) | **0.81 (0.76; 0.86)** | **<0.001** |  |
| Model 1 | **0.94 (0.91; 0.96)** | **<0.001** | 0.96 (0.91; 1.02) | **0.85 (0.80; 0.91)** | **<0.001** |  |
| Model 2 | **0.97 (0.95; 0.99)** | **0.021** | 1.01 (0.95; 1.07) | **0.92 (0.86; 0.99)** | **0.030** |  |
| *Control* |  |  |  |  |  |  |
| Unadjusted | **0.92 (0.90; 0.95)** | **<0.001** | 0.95 (0.89; 1.01) | **0.84 (0.78; 0.89)** | **<0.001** |  |
| Model 1 | **0.94 (0.92; 0.97)** | **<0.001** | 0.97 (0.91; 1.03) | **0.88 (0.83; 0.95)** | **0.001** |  |
| Model 2 | 0.97 (0.95; 1.00) | 0.056 | 1.00 (0.94; 1.07) | 0.95 (0.89; 1.02) | 0.212 |  |
| **Prostate** |  |  |  |  |  | 0.080 |
| *Intervention* |  |  |  |  |  |  |
| Unadjusted | 0.97 (0.91; 1.03) | 0.289 | 1.00 (0.87; 1.15) | 0.91 (0.77; 1.06) | 0.262 |  |
| Model 1 | 0.98 (0.92; 1.04) | 0.533 | 01.02 (0.89; 1.17) | 0.94 (0.80; 1.10) | 0.482 |  |
| Model 2 | 0.96 (0.90; 1.02) | 0.212 | 0.99 (0.86; 1.14) | 0.89 (0.76; 1.05) | 0.203 |  |
| *Control* |  |  |  |  |  |  |
| Unadjusted | 1.04 (0.98; 1.11) | 0.167 | 1.06 (0.92; 1.23) | 1.14 (0.98; 1.34) | 0.096 |  |
| Model 1 | 1.06 (0.99; 1.13) | 0.066 | 1.08 (0.94; 1.25) | **1.18 (1.01; 1.39)** | **0.038** |  |
| Model 2 | 1.05 (0.99; 1.12) | 0.126 | 1.07 (0.93; 1.24) | 1.16 (0.99; 1.37) | 0.070 |  |
| **Colorectal** |  |  |  |  |  | 0.631 |
| *Intervention* |  |  |  |  |  |  |
| Unadjusted | 0.98 (0.88; 1.10) | 0.740 | 1.25 (0.94; 1.66) | 1.04 (0.77; 1.41) | 0.771 |  |
| Model 1 | 0.97 (0.86; 1.08) | 0.561 | 1.22 (0.92; 1.63) | 1.01 (0.74; 1.37) | 0.936 |  |
| Model 2 | 0.99 (0.88; 1.12) | 0.925 | 1.27 (0.95; 1.69) | 1.09 (0.79; 1.49) | 0.586 |  |
| *Control* |  |  |  |  |  |  |
| Unadjusted | 0.92 (0.83; 1.03) | 0.150 | 1.07 (0.82; 1.39) | 0.95 (0.72; 1.26) | 0.731 |  |
| Model 1 | 0.91 (0.82; 1.02) | 0.096 | 1.05 (0.80; 1.37) | 0.93 (0.70; 1.24) | 0.621 |  |
| Model 2 | 0.97 (0.86; 1.08) | 0.546 | 1.12 (0.86; 1.47) | 1.08 (0.80; 1.45) | 0.593 |  |
| **Lung** |  |  |  |  |  | 0.338 |
| *Intervention* |  |  |  |  |  |  |
| Unadjusted | **0.80 (0.75; 0.87)** | **<0.001** | 0.88 (0.74; 1.05) | **0.55 (0.45; 0.68)** | **<0.001** |  |
| Model 1 | **0.81 (0.75; 0.88)** | **<0.001** | 0.87 (0.73; 1.04) | **0.57 (0.46; 0.70)** | **<0.001** |  |
| Model 2 | 1.04 (0.96; 1.12) | 0.368 | 1.15 (0.96; 1.38) | 1.01 (0.82; 1.25) | 0.672 |  |
| *Control* |  |  |  |  |  |  |
| Unadjusted | **0.82 (0.75; 0.88)** | **<0.001** | 0.88 (0.73; 1.07) | **0.64 (0.52; 0.80)** | **<0.001** |  |
| Model 1 | **0.82 (0.75; 0.89)** | **<0.001** | 0.88 (0.73; 1.06) | **0.66 (0.53; 0.81)** | **<0.001** |  |
| Model 2 | 1.03 (0.95; 1.12) | 0.455 | 1.13 (0.94; 1.37) | 1.17 (0.94; 1.47) | 0.131 |  |
| **Ovarian** |  |  |  |  |  | **0.020** |
| *Intervention* |  |  |  |  |  |  |
| Unadjusted | 1.14 (0.95; 1.38) | 0.153 | 1.68 (0.97; 2.92) | 1.68 (0.98; 2.89) | 0.081 |  |
| Model 1 | 1.14 (0.95; 1.38) | 0.155 | 1.68 (0.97; 2.92) | 1.68 (0.98; 2.89) | 0.082 |  |
| Model 2 | 1.17 (0.97; 1.41) | 0.109 | 1.72 (0.99; 3.01) | 1.78 (1.02; 3.09) | 0.055 |  |
| *Control* |  |  |  |  |  |  |
| Unadjusted | 0.89 (0.73; 1.08) | 0.226 | 1.05 (0.64; 1.73) | 0.68 (0.39; 1.16) | 0.147 |  |
| Model 1 | 0.88 (0.72; 1.07) | 0.203 | 1.04 (0.63; 1.70) | 0.66 (0.39; 1.14) | 0.127 |  |
| Model 2 | 0.86 (0.70; 1.05) | 0.138 | 1.01 (0.61; 1.65) | 0.62 (0.36; 1.07) | 0.079 |  |

Model 1 adjusted for age and sex (if applicable). Model 2 adjusted for age, sex (if applicable), race, education, smoking status, cigarette, cigar or pipe smoking. P_trend_ based on midpoint of score tertiles. P_heterogeneity_ represents interaction between score tertile and trial arm.

**Supplementary Figure 1: Prostate, Lung, Colorectal, and Ovarian Cancer Screening Trial data collection timeline**


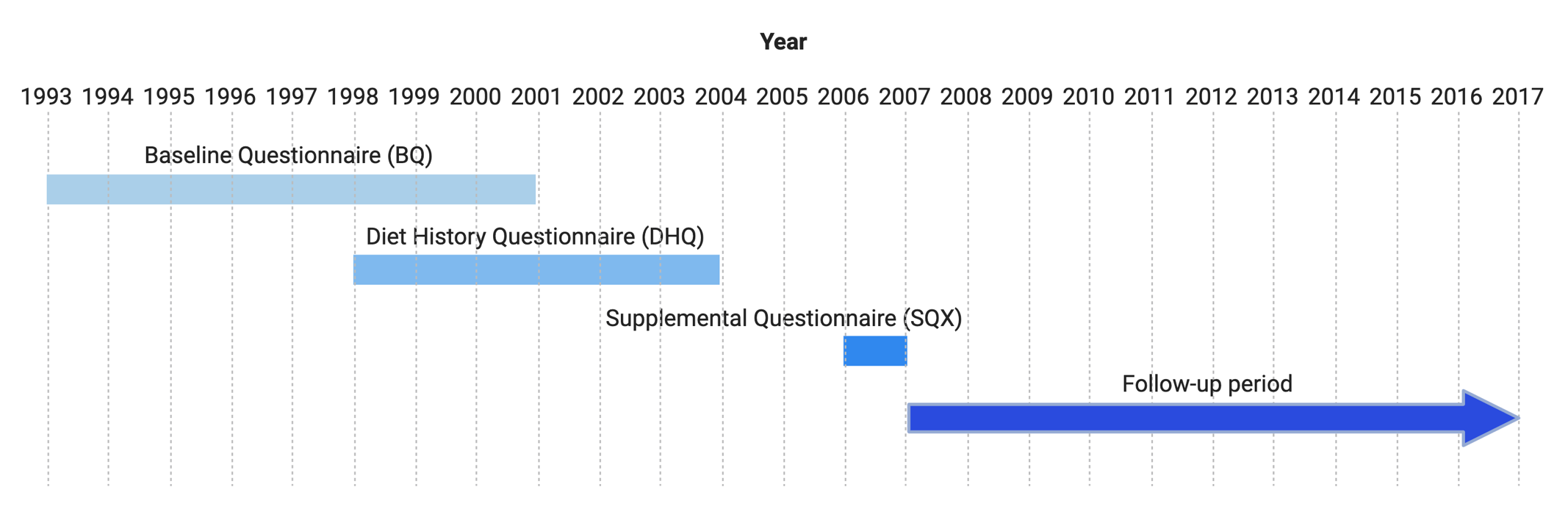


Created in BioRender. Malcomson, F. (2025) <https://BioRender.com/x457iot>

**Supplementary Figure 2:** **Joint effects of 2018 WCRF/AICR Score and smoking status**


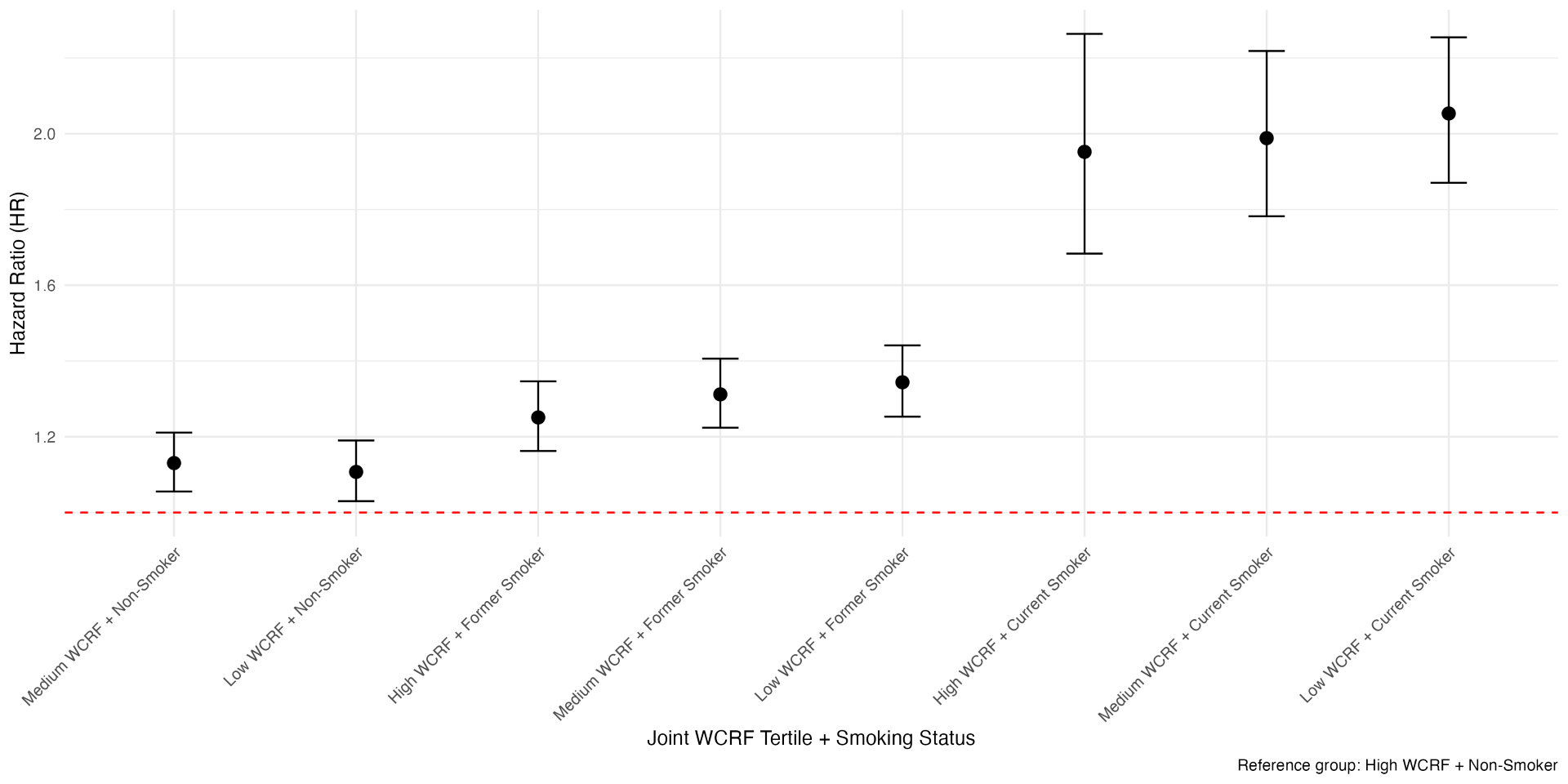


Highest (healthiest) 2018 WCRF/AICR Score tertile participants who were never smokers (dashed red line) was used as the reference group. Model 2 adjusted for age, sex, race, education, cigarette, cigar or pipe smoking.
